# DataColor: unveiling biological data relationships through distinctive color mapping

**DOI:** 10.1093/hr/uhad273

**Published:** 2023-12-21

**Authors:** Shuang He, Wei Dong, Junhao Chen, Junyu Zhang, Weiwei Lin, Shuting Yang, Dong Xu, Yuhan Zhou, Benben Miao, Wenquan Wang, Fei Chen

**Affiliations:** Sanya Institute of Breeding and Multiplication, National Key Laboratory for Tropical Crop Breeding, Hainan University, Sanya 572025, China; School of Tropical Agriculture and Forestry, Hainan University, Haikou 570228, China; Hospital of Stomatology, Guanghua School of Stomatology, Guangdong Provincial Key Laboratory of Stomatology, Sun Yat-Sen University, Guangzhou 510055, China; Department of Biology, Saint Louis University, St Louis, MO 63103, USA; Sanya Institute of Breeding and Multiplication, National Key Laboratory for Tropical Crop Breeding, Hainan University, Sanya 572025, China; School of Tropical Agriculture and Forestry, Hainan University, Haikou 570228, China; Merkle Business Information Consultancy (Nanjing) Co., Ltd, Nanjing 210032, China; Sanya Institute of Breeding and Multiplication, National Key Laboratory for Tropical Crop Breeding, Hainan University, Sanya 572025, China; School of Tropical Agriculture and Forestry, Hainan University, Haikou 570228, China; Shenzhen Branch, Guangdong Laboratory for Lingnan Modern Agriculture, Genome Analysis Laboratory of the Ministry of Agriculture and Rural Affairs, Agricultural Genomics Institute at Shenzhen, Chinese Academy of Agricultural Sciences, Shenzhen 518120, China; State Key Laboratory of Rice Biology & Breeding, Zhejiang Provincial Key Laboratory of Crop Germplasm, The Advanced Seed Institute, Zhejiang University, Hangzhou 310058, China; College of Ocean and Earth Sciences, Xiamen University, Xiamen 361102, Fujian, China; Sanya Institute of Breeding and Multiplication, National Key Laboratory for Tropical Crop Breeding, Hainan University, Sanya 572025, China; School of Tropical Agriculture and Forestry, Hainan University, Haikou 570228, China; Sanya Institute of Breeding and Multiplication, National Key Laboratory for Tropical Crop Breeding, Hainan University, Sanya 572025, China; School of Tropical Agriculture and Forestry, Hainan University, Haikou 570228, China

## Abstract

In the era of rapid advancements in high-throughput omics technologies, the visualization of diverse data types with varying orders of magnitude presents a pressing challenge. To bridge this gap, we introduce DataColor, an all-encompassing software solution meticulously crafted to address this challenge. Our aim is to empower users with the ability to handle a wide array of data types through an assortment of tools, while simultaneously streamlining parameter selection for rapid insights and detailed enhancements. DataColor stands as a robust toolkit, encompassing 23 distinct tools coupled with over 600 parameters. The defining characteristic of this toolkit is its adept utilization of the color spectrum, allowing for the representation of data spanning diverse types and magnitudes. Through the integration of advanced algorithms encompassing data clustering, normalization, squarified layouts, and customizable parameters, DataColor unveils an abundance of insights that lay hidden within the intricate relationships embedded in the data. Whether you find yourself navigating the analysis of expansive datasets or embarking on the quest to visualize intricate patterns, DataColor stands as the comprehensive and potent solution. We extend the availability of DataColor to all users at no cost, accessible through the following link: https://github.com/frankgenome/DataColor.

## Introduction

Plant research has generated various types of data from sources such as genomics [1], phenomics [2], metabolomics [3], single-cell omics [4], leading to the rise of big data research in this field. Visualizing different types and magnitudes of data poses significant challenges. One effective approach is to use multiple algorithms and colors to visualize the data. For instance, heatmaps are powerful data visualization tools that effectively display and cluster data. By employing a limited number of spectral colors, heatmaps allow users to easily distinguish different data clusters and observe their distribution patterns [5–10]. This facilitates the identification of trends and patterns in the data, enabling more informed decision-making. Heatmaps have gained popularity in gene expression studies and genome assembly as they facilitate the exploration of data correlations [11]. Furthermore, the electronic Fluorescent Pictograph (eFP) browser serves as a visualization toolkit, providing an intuitive means to study spatiotemporal gene expression in individual organisms [12]. Despite the increasing demand for using colors to visualize diverse types and magnitudes of biological data, comprehensive software specifically designed for this purpose remains lacking. As a result, researchers face challenges in effectively interpreting and communicating their data.

**Figure 1:**
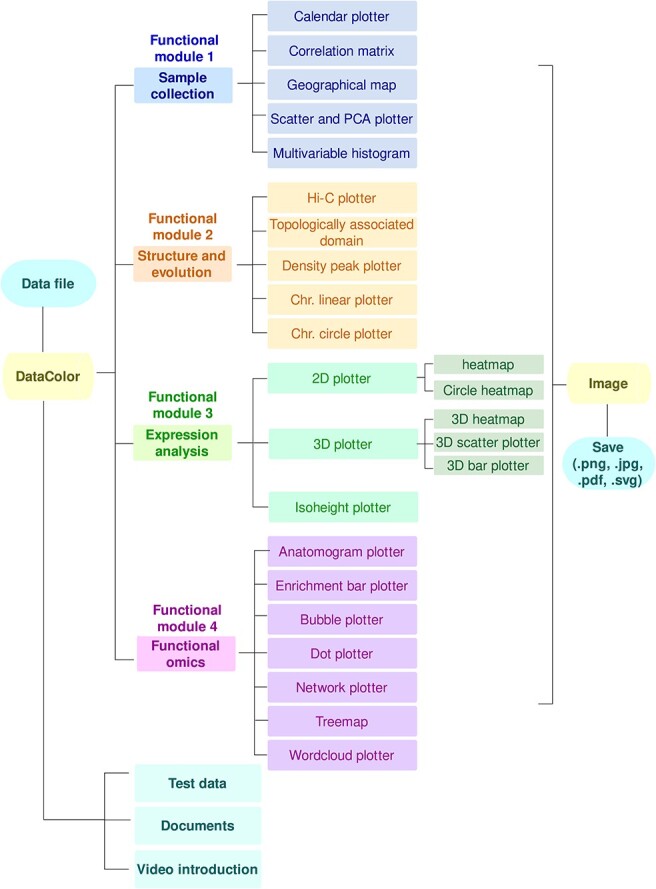
Workflow Chart of DataColor Software. The comprehensive DataColor workflow comprises four distinct modules: ‘Sample Collection’, ‘Structure and Evolution’, ‘Expression Analysis’, and ‘Functional Omics’, totally encompassing a collection of 23 tools. Embedded within the software are provisions for test data, documents, and video introductions, augmenting the user experience.

**Figure 2:**
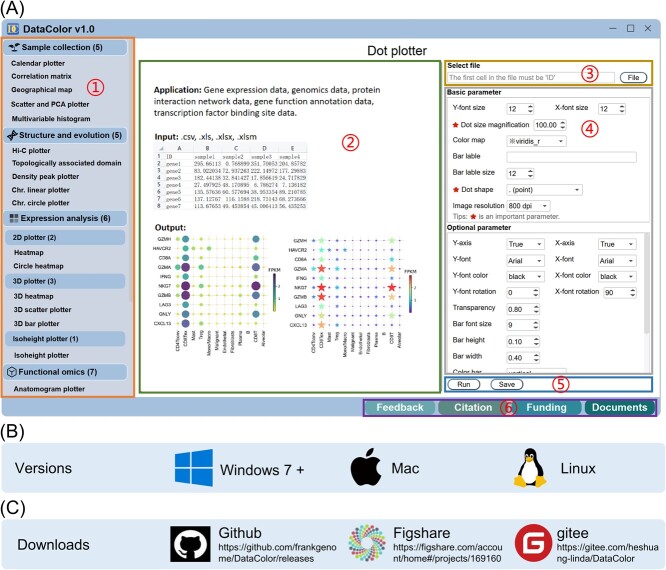
The DataColor Interface. (**A**) The DataColor interface showcases a division into six primary sections, each denoted by a number within a circle, highlighting the distinct functional components within the interface. Positioned on the left, the toolbox houses an assortment of 23 diverse tools. (**B**) Notably, DataColor is designed to cater to three major platforms—Windows, Mac, and Linux—ensuring cross-platform accessibility and utility. (**C**) DataColor is available at three public databases.

The rapid pace of technological innovation has led to the development of various bioinformatics software programs, pipelines, and packages for creating images. For example, heatmaps are very popular and could be plotted by gpplot2 in R [13], Pheatmap [14], aheatamp [15], Corrplot, ComplexHeatmaps [16], Heatmap3 [17], D3.js [18], Matplotlib [19], Seaborn for python [20], SPSS and SAS [21], Matlab (https://www.mathworks.com). Programming skill is typically required to use these tools effectively. Several tools, including ClustVis [22], Perseus [23], TBtools [24] and MetaboAnalyst 5.0 [25] and Hiplot [26], offer comprehensive visualization capabilities beyond heatmap generation. Despite these advancements, heatmap tools still have some deficiencies. For example, they may not offer enough parameters or sufficient richness in the heatmaps produced. Additionally, it can be challenging for users to conveniently and quickly select parameters and debug heatmaps, and the resulting heatmaps may not always meet complex requirements. Despite these challenges, heatmap tools remain valuable resources for researchers seeking to visualize complex data sets. Advancements in technology and ongoing developments in heatmap tools will undoubtedly continue to improve the quality and accessibility of heatmap visualization in the years to come.

The importance of retrieving information from big data in a comprehensive, fast, easy, and intuitive way is increasing rapidly. To address this need, we have developed the DataColor tool, which employs color spectrum to represent different magnitudes and different kinds of data, to serve as a one-stop-shop for analysing various kinds of omics data for a variety of purposes. We hope that with DataColor, accessing and visualizing big data will never be simpler or more efficient.

## Results

### Software introductions

DataColor (Fig. 1) aims at utilizing a rich color spectrum to represent diverse data and facilitate the identification of correlations between datasets (Fig. S1, see online supplementary material). The installation packages, source codes, user manual, test data, and all relevant files have been uploaded to public databases, including GitHub (https://github.com/frankgenome/DataColor), figshare (https://figshare.com/account/home#/projects/169160), and gitee (https://gitee.com/heshuang-linda/DataColor). DataColor presents an intuitively designed software tailored for user convenience, eliminating the need for programming expertise. The step-by-step operational process on DataColor’s homepage (Fig. 2) is as follows: 1. ‘Tool or Method Selection’: Begin by choosing the desired tool or method. 2. ‘File Input’: Import files effortlessly by either clicking the ‘File’ button or simply dragging and dropping the file into the interface. 3. ‘Parameter Configuration’: Customize settings by selecting the appropriate parameters for your task. 4. ‘Execution’: Initiate the process by clicking the ‘Run’ button. 5. ‘Visual Display’: View the generated image as a result of the performed operation. 6. ‘Documentation Assistance’: For further guidance, refer to the comprehensive help documents. All these actions are seamlessly executed by clicking the corresponding buttons, ensuring an uncomplicated user experience. To enhance the quality of visuals, users can generate and export heatmaps by conveniently right clicking the ‘Save’ option. Notably, DataColor offers a spectrum of four resolutions, ranging from 300 to 1000 dpi, to ensure exceptional graphic output. Furthermore, DataColor supports a versatile range of output formats, including scalar and scalable vector graphics (SVG), portable network graphics (PNG), joint photographic experts group (JPEG), and portable document format (PDF). This diverse format support guarantees compatibility and flexibility for various project requirements.

DataColor comprises 23 tools, unlike previous comprehensive tools such as TBtools or HiPlot (Table 1), all employ color spectrum to represent various types and magnitudes of data to serve the four modules in plant research. The first module, ‘Sample collection’, facilitates the visualization of sampling data, including, a ‘Calendar plotter’, a ‘Correlation matrix’, a ‘Geographical map’, a ‘Scatter and PCA plotter’, and a ‘Multivariable histogram’. The second module, ‘Structure and evolution’, promotes the visualization of chromosome structures, genes, molecular markers, repetitive sequences, and other data. It includes a ‘Hi-C tool’, a ‘Topologically associated domain (TAD)’ tool, a ‘Chr. linear plotter’, ‘Chr. circle plotter’, and facilitating structural genomics research. Additionally, a ‘Density peak plotter’ is available for gene *K*s distribution studies, aiding genome duplication research. The third module, ‘Expression analysis’, supports gene expression profiling research. It includes two two-dimensional (2D) plotters, three three-dimensional (3D) plotters, and an isoheight plotter. The fourth module, ‘Functional omics,’ assists in visualizing multi-omics data, revealing the regulatory mechanisms of biological processes. It comprises an ‘Anatomogram plotter’, an ‘Enrichment bar plotter’, a ‘Bubble plotter’, a ‘Dot plotter’, a ‘Network plotter’, a ‘Treemap’ tool, and a ‘Wordcloud plotter’. DataColor’s diverse set of tools, with over 600 adjustable parameters, user-friendly interface, and detailed usage instructions make it the ideal solution for visualizing different types and magnitudes of biological data using color.

**Table 1:** Comparison of DataColor with other related tools.

Items	DataColor (This study)	HiPlot	ImageGP	TBtools	CoolBox	ggplot2
Tools or Functions						
Target or scope	Mainly plant sciences	Mainly biomedicine	Biological data plotter	Comprehensive tool	Mainly Hi-C data plotter	Comprehensive data plotter
Novel 3D plotting and iso-height plotting	√	×	×	×	×	×
7 clustering methods and 22 metric	√	×	×	×	×	√
Different tools can be compared on the same interface	√	×	×	√	×	×
Hi-C plotter and TAD plotter	√	√	√	×	√	√
Chr. circle plotter	√	√	×	√	×	×
Size scaling parameters for nodes and bubbles	√	×	×	×	×	×
The node plotter and scatter plotter offer 23 different node types	√	×	×	×	×	×
Calendar plotter	√	√	×	×	×	×
Treemap plotter	√	√	×	×	×	×
Network plotter	√	√	×	×	×	×
The Network Plotter provides six layout options	√	√	×	×	×	×
Geographical map	√	√	×	×	×	×
Wordcloud plotter	√	√	×	×	×	×
Density peak plotter	√	√	×	×	×	×
Circle heatmap	√	×	×	√	×	×
Chr. density plotter	√	×	×	×	×	×
The output images have multiple resolution parameters	Four resolutions	×	×	Two resolutions	×	×
No need to master programming languages	√	√	√	√	√	×
User interface						
Input file format reminder	√	×	×	×	×	×
Each tool’s usage illustration includes both an input file diagram and an output file diagram	√	√	×	×	×	×
Environment and platform						
Supports Windows, Mac, and Linux operating systems	√	√	×	Supports Windows and Mac operating systems	×	√
Available for download on GitHub, figshare, and Zenodo	√	×	×	×	×	×
Others						
No registration required	√	×	√	√	√	√
Open source	All	partly	×	partly	partly	All
Not affected by network restrictions	√	√	×	√	√	√
Supports local execution	√	√	×	√	√	×

**Figure 3:**
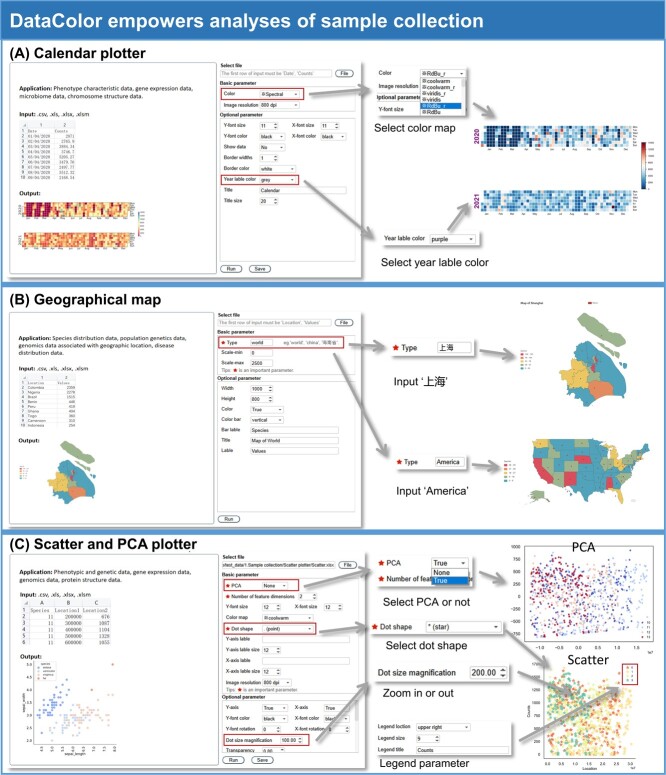
DataColor empowers the analyses of sample collection. (**A**) DataColor provides the calendar plotter for visualization of recording sampling issues. (**B**) DataColor allows the labelling of sampling sites on the global map or local map. (**C**) DataColor helps analyses on population samples using scatter plot or principal component analysis (PCA).

**Figure 4:**
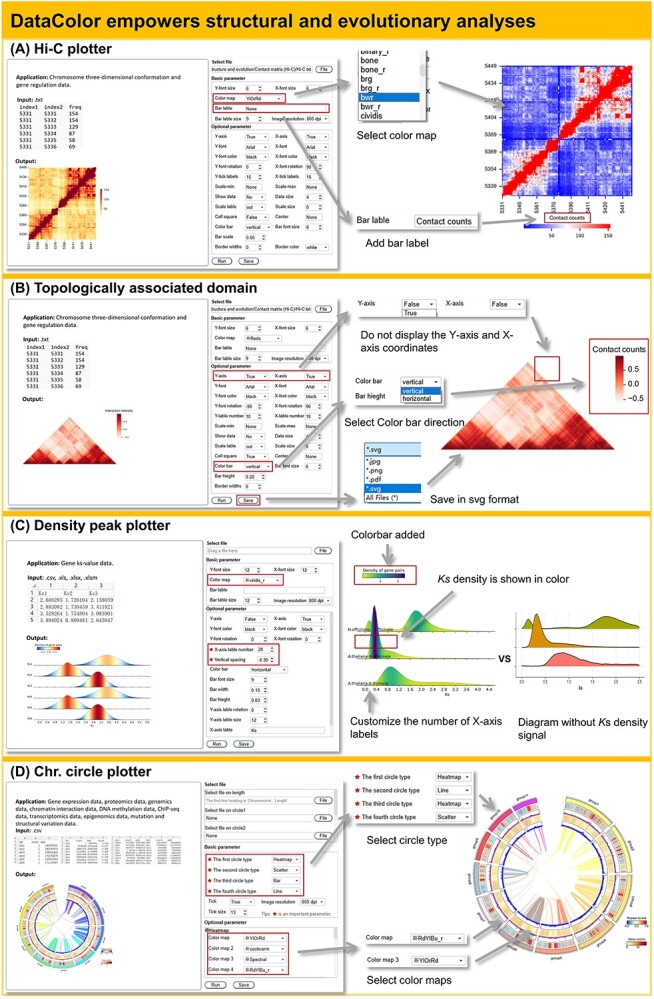
Introduction to the Tools in the ‘Structure and Evolution’ Module. **(A)** DataColor facilitates the visualization of Hi-C data through a diverse range of color options. Each colorbar’s label is customizable within the DataColor interface. **(B)** Addressing the needs of 3D genomics, DataColor empowers users to visualize topological domain data, offering compatibility with several output formats, including jpg, png, pdf, and svg. **(C)** In a notable innovation, the density peak plotter is meticulously designed to unveil previously overlooked density signals, shedding new light on data patterns. (**D**) DataColor provides the visualization of genome details by chr. Circle plotter.

## Integrated tools

### Sample collection and analyses

Large-scale sampling data form the foundation of scientific research, and DataColor is committed to visualizing this diverse data. The ‘Calendar plotter’ is a data visualization tool used for feature analysis of time-series data (Fig. 3A). It enables the recording of plant-related phenotypic traits over time, facilitating the analysis of temporal changes in the data. The ‘Correlation matrix’ tool uses a popular approach to display the correlations between multiple variables in the dataset, revealing the relationships between genes, samples, and replicates (Fig. S2A, see online supplementary material). The ‘Geographic map’ tool allows users to display geographic data on maps using colors. These maps cover 200 countries and regions worldwide. Specifically, the map of China can display data from province to city to county/district levels. It can be used to statistically represent plant samples from different regions, such as a ‘Geographic map’ illustrating the distribution of cassava varieties from various countries in the cassava germplasm resources (Fig. 3B). The ‘Scatter and PCA plotter’ represent the distribution of data points on a Cartesian coordinate plane, making them suitable for analysing sample population data and observing distribution patterns and trends (Fig. 3C). The ‘Multivariable histogram’ tool is generally suitable for plotting data with multiple variables or features to study the relationships and distributions between them (Fig. S2B, see online supplementary material).

**Figure 5:**
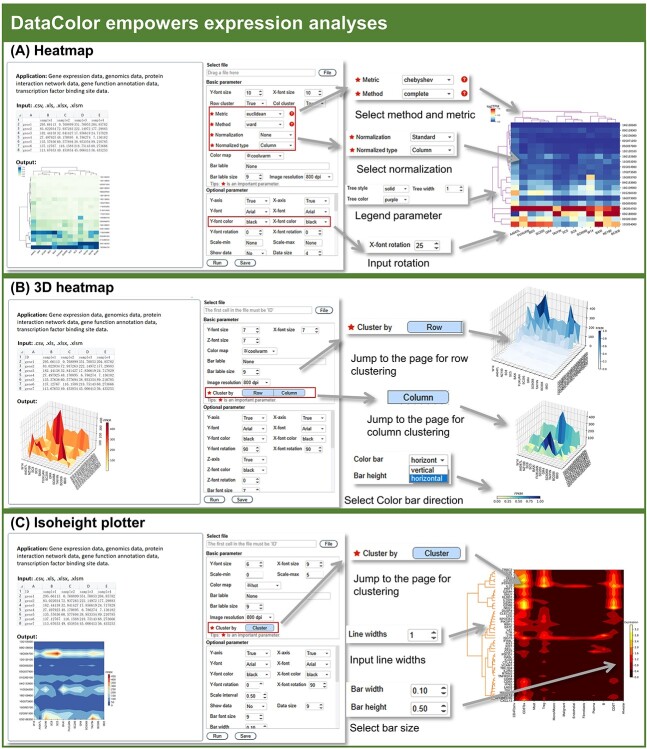
DataColor empowers visualization of gene expressions. (**A**) The ‘Heatmap’ tool provides a series of parameters for visualization of gene expressional data. (**B**) The ‘3D heatmap’ tool could show the expressional data in three dimensions. (**C**) The ‘Isoheight plotter’ shows the expressional data with melting boundaries.

### Structure and evolution

Exploring the structure and evolution at the genomic level is a hot topic in research, and DataColor provides tools for this purpose. The Hi-C tool and TAD tool are offered to facilitate the visualization and analysis of data correlations. Hi-C and TAD graphs can be generated from preprocessed and transformed Hi-C data, and they can be adjusted using a series of parameters to aid data visualization and correlation analysis. For instance, Hi-C scaffolding and a topologically associated domain analyses are useful in displaying part of the cassava chromosome (Fig. 4A and B). The ‘Density peak plotter’ can be used to compare selection pressure and evolutionary rates between different species or genes. It has important applications in studying selection pressure and evolutionary rates of genes, as well as genome duplication (Fig. 4C). Compared to conventional *K*s distribution plots, the ‘Density peak plotter’ provides a better understanding of the precise distribution density of Ks, allowing for a more detailed resolution of peak characteristics. The ‘Chr. density plotter’ could display the distribution of molecular markers, GC content, gene distribution, repetitive sequences, and more, on the chromosome (Fig. S3, see online supplementary material). The ‘Chr. circle plotter’ is a sophisticated drawing tool capable of showcasing various types of visualizations, such as heatmaps, bar graphs, line charts, and scatter plots (Fig. 4D). It is applicable to diverse data sets including gene expression, proteomics, genomics, chromatin interaction, and more.

### Expression analysis

Expression analysis specifically caters to the exploration of data correlations and is particularly useful for gene expression profiling studies. DataColor offers users a variety of heatmap drawing tools, providing powerful and intuitive means to analyse and visualize complex datasets. Furthermore, it has developed multiple 3D plotters, which are expected to facilitate correlation mining at different depths of the data. The 2D plotters include normalized and clustered plotters. The ‘Heatmap’ tool generates user-friendly heat maps, applicable in scenarios where clustering is not required but a direct representation of the heatmap is needed, as well as in clustered heatmap scenarios (Fig. 5A). This tool incorporates standardization parameters, divided into standard and Z-score options, encompassing both column and row standardization to alleviate differences in data magnitude. Clustering algorithms are primarily utilized in unsupervised learning and structured data analysis. Clustering algorithms are primarily used for unsupervised learning and structured data analysis. The ‘Circle heatmap’ tool presents heatmap data in a circular format and introduces clustering functionality (Fig. S4, see online supplementary material). 3D heatmaps are relatively rare in biological data analysis. To fill this gap, DataColor has developed five 3D plotters, including ‘3D heatmap’ (Fig. 5B), ‘3D bar plotter’ (Fig. S5A, see online supplementary material), and ‘3D scatter plotter’ (Fig. S5B, see online supplementary material). Each clustering tool supports seven types of clustering methods, 22 distance metrics, various data optimizations, and parameters to aid in visualizing and analysing complex data in 3D. The ‘3D scatter plotter’ and ‘3D bar plotter’ allow for the examination of data correlations from multiple dimensions. The ‘Isoheight plotter’ tool offers two modes: normal and cluster, both presenting data in 2D and simulating a 3D scene by connecting neighboring points and similar data into a loop. The ‘Isoheight plotter (cluster)’ tool (Fig. 5C) provides seven clustering methods and 22 distance metrics. These complex heatmaps are particularly useful for projects involving large-scale biological data [16]. To highlight the usefulness of these tools in botanical research, we used the expression levels of the C4 pathway in photosynthesis-carbon fixation and the dark reaction for 13 cassava varieties to create 2D and 3D heatmaps.

### Functional omics

**Figure 6:**
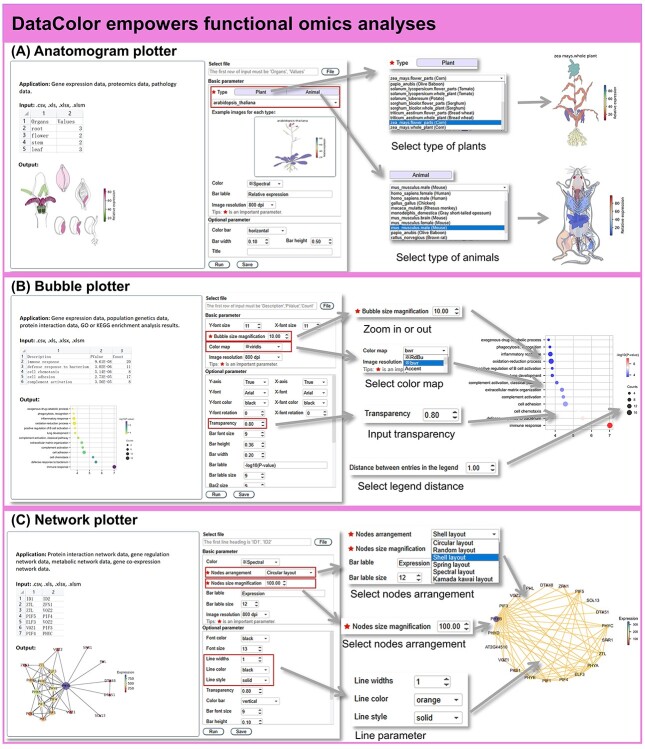
DataColor empowers the functional omics studies. (**A**) The anatomograph plotter shows the gene expression on 17 model species including plants and animals. (**B**) The bubble plotter facilitates visualization of the GO and KEGG annotations. (**C**) The network plotter shows the gene network using enrichment nodes.

Functional omics aims to serve the analysis and exploration of plant multi-omics data. Visualizing data related to multicellular organism organs poses challenges, but the ‘Anatomogram plotter’ addresses this by providing representations of 17 representative plants or plant organs, such as Arabidopsis, rice, maize, *etc* (Fig. 6A). By assigning different colors to different organs (representing varying expression levels), users can easily detect gene expression differences and correlations between organs. The ‘Enrichment Bar Plotter’ visually represents data differences and analyses the relationships between data through the positions, heights, and colors of the bars in a bar chart (Fig. S6A, see online supplementary material). The ‘Bubble plotter’ explores data correlations through the position, size, and color of bubbles. The diversity in shapes enhances the visual appeal of the heatmap and allows for more detailed data representation (Fig. 6B). The ‘Dot plotter’ tool offers 23 different point shapes, including dots, diamonds, and stars, providing a rich graphical display for the heatmap (Fig. S6B, see online supplementary material). The ‘Bubble plotter’ connects nodes with one or more lines, where the size of the nodes depends on their correlations. With six different node layout methods, including random, grid, and algorithmic layouts, this tool offers a powerful means to display differential co-expression gene networks. Relying on ‘Network plotter’, we use module data from the co-expression gene network in cassava under drought treatment to highlight this feature (Fig. 6C). These layouts enable researchers to effectively visualize and explore the connections between different genes, providing valuable insights into potential biological mechanisms. The ‘Treemap’ tool relies on using nested rectangles to display data in a hierarchical structure (Fig. S6C, see online supplementary material). ‘Enrichment Bar Plotter’, ‘Bubble plotter’ and ‘Treemap’ are particularly useful when dealing with small amounts of data, such as Gene Ontology (GO) and Kyoto Encyclopedia of Genes and Genomes (KEGG) enrichment data. The ‘Wordcloud plotter’ represents text data using various word clouds in a colorful graphic manner (Fig. S7, see online supplementary material). Users can customize the background silhouette to create maps of different shapes. This tool is widely used for analysing expression values in histology research, GO or KEGG enrichment, and other similar applications. For instance, data from GO enrichment analysis of drought-treated cassava leaves compared to control leaves can be visualized using ‘Treemap’, ‘Bubble Plotter’, and ‘Wordcloud Plotter’.

## Discussion

In an era marked by an escalating influx of biological data, encompassing a spectrum of multi-omics information spanning genomics [27–31], transcriptomics [32–36], metabolomics [34], phenomics [37, 38], and histological data [39, 40], the imperative to visualize this data stands as a paramount challenge for bioinformatics researchers. Their objective revolves around unraveling relationships and unearthing pivotal insights from this vast trove of information. For instance, recent advancements in omics literature have harnessed various visualization techniques to depict intricate connections. Heatmaps have proven invaluable for elucidating associations between expressions [37], while non-clustered 2D heatmaps have artfully illustrated Hi-C auxiliary chromosome formations [41]. Bubble maps, on the other hand, have been adeptly employed to showcase Gene Ontology (GO) enrichments [42]. Additionally, non-clustered heatmaps have emerged as a compelling choice to convey intricate relationships [43]. As the data landscape continues to burgeon, the role of visualization remains pivotal in translating raw data into comprehensible insights. It is through these visual representations that the intricate tapestry of biological intricacies is brought to light, facilitating a deeper understanding of biological systems and phenomena.

With the increasing amount of biological data, various data visualization tools have been developed, but a complete tool suite to complete a range of mapping tasks is still lacking. Moreover, existing tools generally suffer from insufficient parameters, imperfect functions, and complex procedures. Compared to other related software, DataColor stands out as the only one that exclusively focuses on utilizing colors to represent diverse types and magnitudes of data. It employs algorithms such as data clustering, normalization, squaring, and parameter range adjustments to reveal data correlations, facilitating the integration of various types of big biological data for research. In contrast to other language libraries like ggplot2 [13], D3.js [18], and Matplotlib [44], DataColor is designed for ease of use and does not require any programming knowledge, making it accessible to a wider range of users. While TBtools [24] is used for local purposes and Hiplot [26] for web usage, DataColor supports both local use and as a software package for Windows, Mac, and Linux, catering to diverse user needs. It places a strong emphasis on data visualization, offering 23 different types of tools and 600 parameters. DataColor integrates data analysis and visualization steps into a single workflow, significantly enhancing operational convenience.

Additionally, DataColor provides a series of unique and innovative tools. Compared to Heml [45], a web-based heatmap tool, DataColor offers a broader range of heatmap types and parameters. DataColor stands out for its innovation compared to other software, particularly in the extension of structural genomics applications, innovation in 3D tools, introduction of background tools, and the richness and completeness of parameters. It proves to be highly valuable in analysing various aspects of plant histological data. We believe that DataColor will become a valuable tool for visualizing and analysing big biological data.

However, the current DataColor v1.0 is limited in two ways. First, relying on Python as the architecture may create challenges when processing millions of data. Second, DataColor currently lacks interactive interfaces, which is an area that we plan to focus on in future versions.

## Materials and methods

### Development process

The creation of DataColor was meticulously undertaken utilizing Python v3.8, encompassing the realization of a user-friendly graphical interface (GUI) facilitated by PyQt5. In order to cater to a wide user base, DataColor is accompanied by tailored installation packages designed for seamless integration with the three predominant operating systems: Windows, Macintosh, and Linux.

### Algorithms employed

Within DataColor, a data normalization technique is harnessed, where the ‘Normalization (Column/Row)’ parameter employs a mean normalization equation. This equation calculates the mean and standard deviation of the original dataset to effectively normalize the data, enhancing its interpretability and analytical accuracy. The equation is:

$$ {D}_{mn}=\frac{D-\overline{D}}{\sqrt{\frac{1}{N}{\sum}_{i=1}^n{\big(D-\overline{D}\big)}^2}} $$

where${D}_{mn}$ denotes the normalized value, and$D$ denotes the original value of the data ($D$ >0), and$\overline{D}$ denotes the mean value of the data. The processed data conform to the standard normal distribution, i.e., the mean value is 0 and the standard deviation is 1.

Standard parameter uses the Standard algorithm to normalize this dimension for rows or columns, i.e., each row or column is subtracted from the data minimum and divided by the data maximum. The formula is:

$$ {D}_{str}=\frac{D-{D}_{min}}{D_{max}} $$

where${D}_{str}$ denotes the Standard normalized value, the${D}_{min}$ and ${D}_{max}$ denote the minimum and maximum values, respectively, of all$D$ the minimum and maximum values of (${D}_{max}>{D}_{min}$ and${D}_{min}$ > 0). The Z-score parameter applies the mean normalization equation, and the standardization principle of the Z-score is that the Z-score transforms two or more data sets into unitless Z-score scores by (x-μ)/σ, which makes the data standardized, improves data comparability, and weakens data interpretation.

The ‘Network plot’ function is not set in dynamic form, but DataColor also uses six layout algorithms to optimize the spatial layout of the network nodes. The layout form of ‘Spring layout’ uses the Fruchterman-Reingold algorithm to arrange the nodes to reduce the intersection of edges in the layout and keep the length of edges as consistent as possible. The node layout of ‘Spectral layout’ is arranged according to the Laplace eigenvector, which reflects a potential applied on node ‘i’ and in which direction this potential can flow more smoothly to other nodes. ‘Kamada–kawai layout’ is a Kamada–Kawai algorithm using force-oriented layout, which incorporates the concept of ideal distance between non-adjacent nodes, where the ideal distance between two nodes is proportional to the length of the shortest path between them.

The ‘Treemap’ function employs the Squarified algorithm, which aims to create rectangles that closely resemble squares and have a more balanced aspect ratio. This algorithm arranges the child nodes in descending order of size, starting with the node that has the largest weight. The nodes are then filled from left to right or bottom to top, following the principle of starting along the shortest edge first, immediately to the left or bottom. After each child node is filled, the algorithm compares the average aspect ratio of the first to n-1 rectangles. It does this by either inserting the new rectangle into the existing rows and columns, or creating a new row or column, using a peer-to-peer insertion method. The algorithm then selects the filling method that results in the lower average aspect ratio for the nth child node.

### Parameter configuration

DataColor boasts an extensive array of customizable parameters designed to cater to diverse analytical needs. This includes the implementation of seven clustering methods and 22 distance metrics, ensuring versatile exploration of data relationships. The seven clustering methods encompass ‘average’, ‘single’, ‘complete’, ‘weighted’, ‘centroid’, and ‘median’. In parallel, the distance function offers a comprehensive spectrum of options, encompassing ‘braycurtis’, ‘canberra’, ‘chebyshev’, ‘cityblock’, ‘correlation’, ‘cosine’, ‘dice’, ‘euclidean’, ‘hamming’, ‘jaccard’, ‘jensenshannon’, ‘kulczynski1’, and ‘mahalanobis’, among others. The heatmap’s color function within DataColor predominantly harnesses cmap colors, wherein the cmap parameter facilitates dynamic color mapping. Impressively, the cmap parameter encompasses 166 distinct types, thoughtfully categorized into Sequential, Diverging, Qualitative, and Miscellaneous colormaps. This comprehensive selection empowers users to effectively portray data nuances with precision and clarity.

## Acknowledgements

This work was supported by the National Natural Science Foundation of China (32472614), National Natural Science Foundation of China-CG joint foundation (3181101517), Hainan Province Science and Technology Special Fund (ZDYF2023XDNY050). Authors thank the anonymous reviewers for their invaluable comments and suggestions.

## Author contributions

 Fei Chen and Wenquan Wang designed and led this project. Shuang He and Junhao Chen wrote the codes. Shuang He, Wei Dong, Junyu Zhang, Weiwei Lin, Shuting Yang, Dong Xu, Yuhan Zhou, and Benben Miao participated in software improvement. Shuang He and Fei Chen wrote the draft manuscript. W.W., Shuang He, and Fei Chen discussed and revised the manuscript. All authors have read and agreed the final manuscript.

## Data availability

The software, user documents, and test data are available at GitHub (https://github.com/frankgenome/DataColor), gitee (https://gitee.com/heshuang-linda/DataColor), and figshare (https://figshare.com/account/home#/projects/169160).

## Conflict of interest statement

The authors declare that they have no conflict of interest.

## Supplementary data

Supplementary data is available at *Horticulture Research* online.

## Supplementary Material

Web_Material_uhad273
