# Supplementary material for: DataColor: unveiling biological data relationships through distinctive color mapping
Source: Hortic Res. 2023 Dec 21;11(2):uhad273. doi: 10.1093/hr/uhad273 (PMC10852383; doi:10.1093/hr/uhad273)
Supplement: Web_Material_uhad273 [file web_material_uhad273.zip › Supplementary Figures.docx]

**Supplementary Figures**


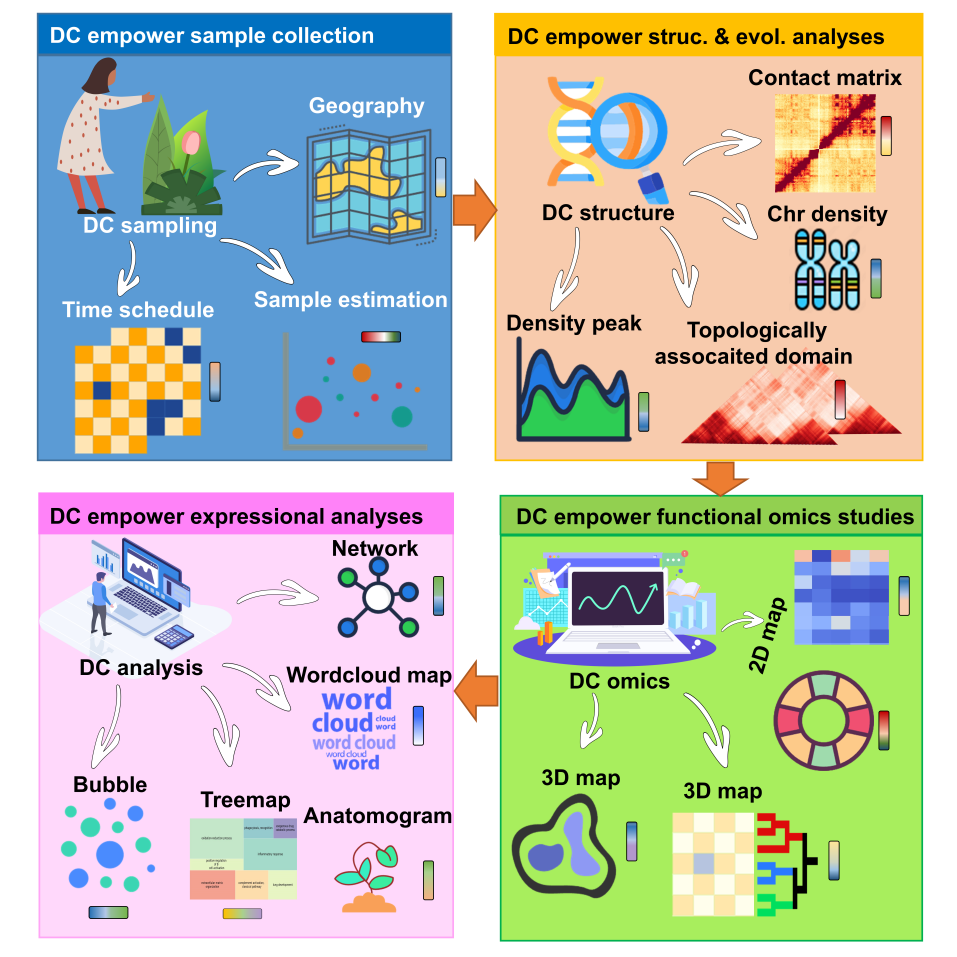


**Supplemental Figure 1. The four major modules of DataColor tools.** DataColor includes 23 tools, serving four processes of experimental research. The first category is "Sample collection", the second category is "Structure and evolution", the third category is "Expression analysis", and the fourth category is "Functional omics".**
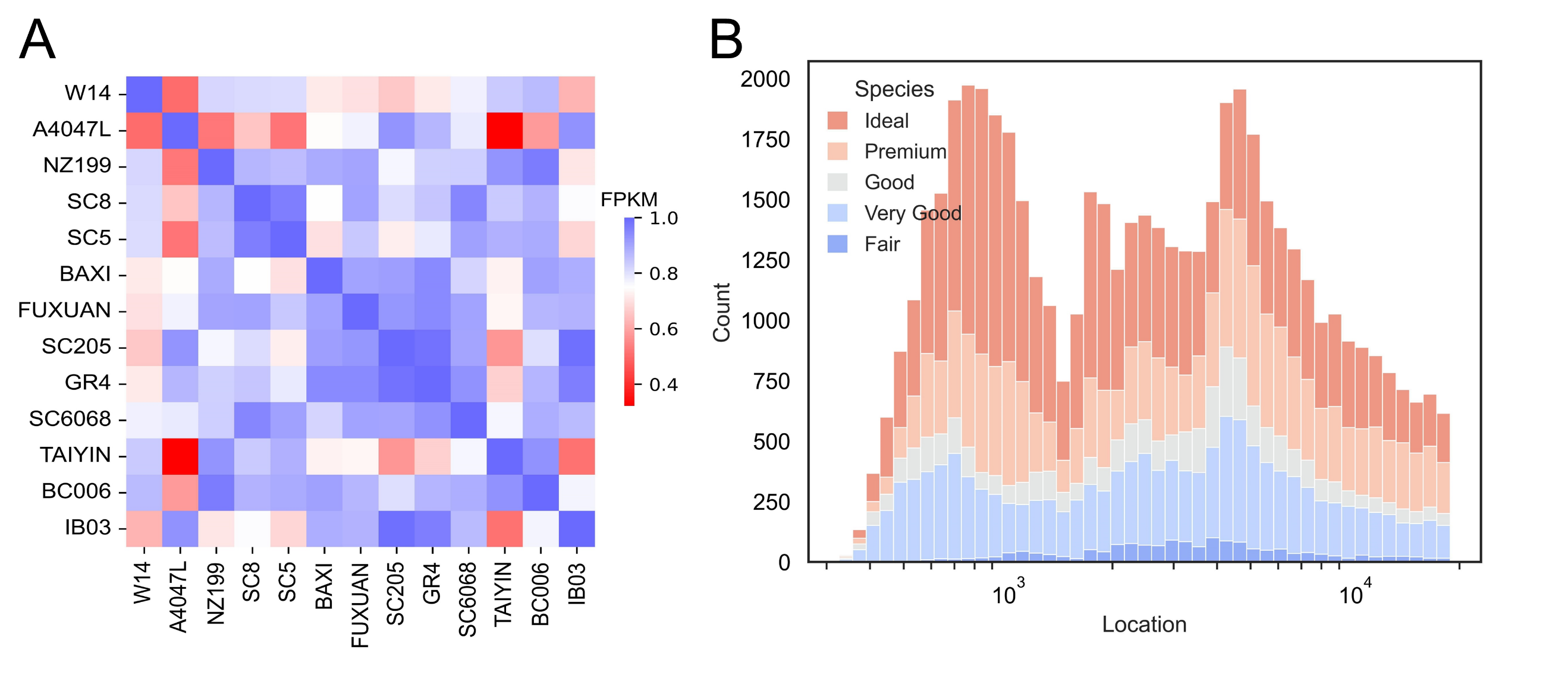
Supplemental Figure 2. Correlation matrix plot and multivariable histogram produced by DataColor.**


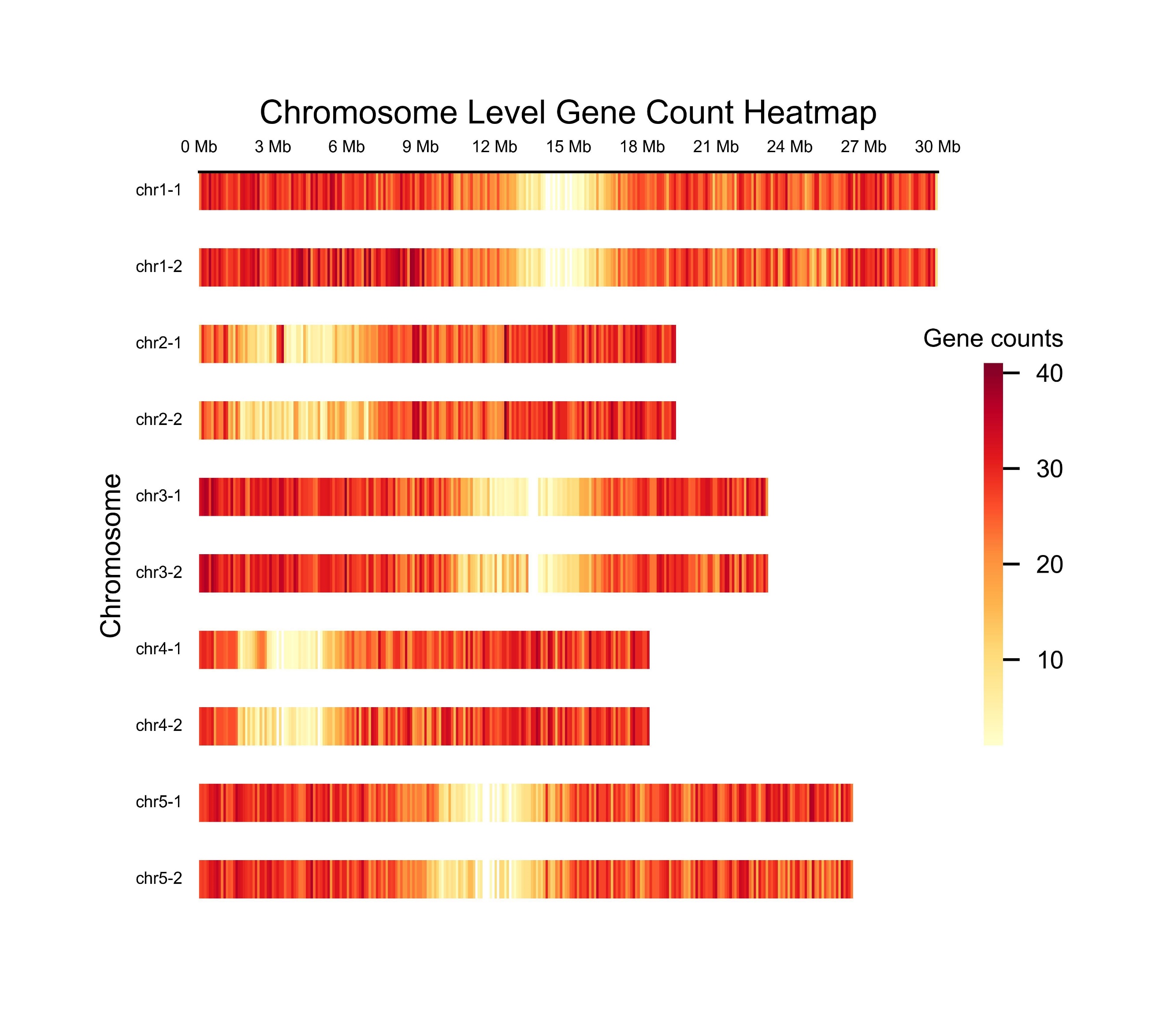


**Supplemental Figure 3. Chr. linear plot produced by DataColor.**

**
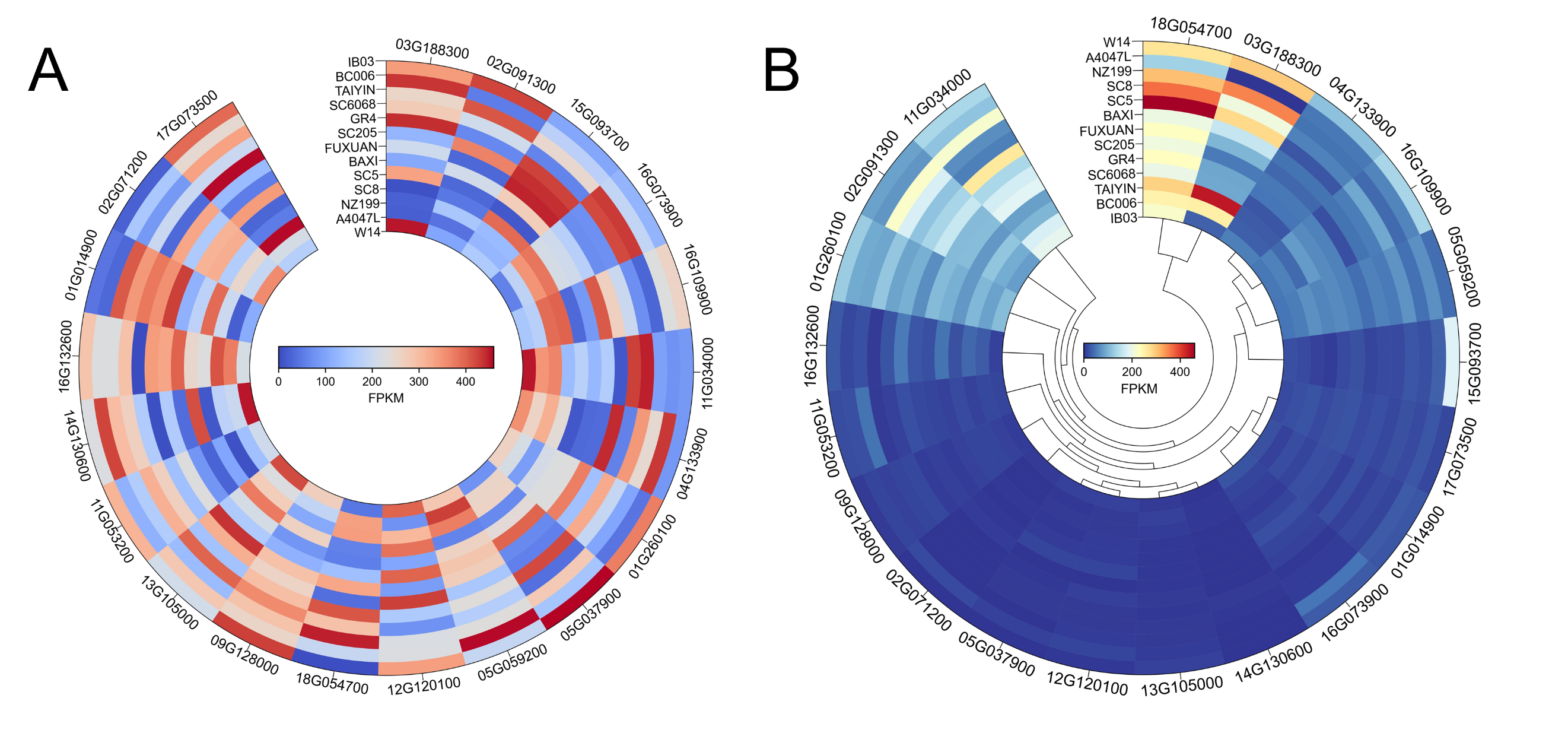
**

**Supplemental Figure 4. Circle heatmap produced by DataColor.** (A) Circle heatmap. (B) Circle heatmap (cluster).

**
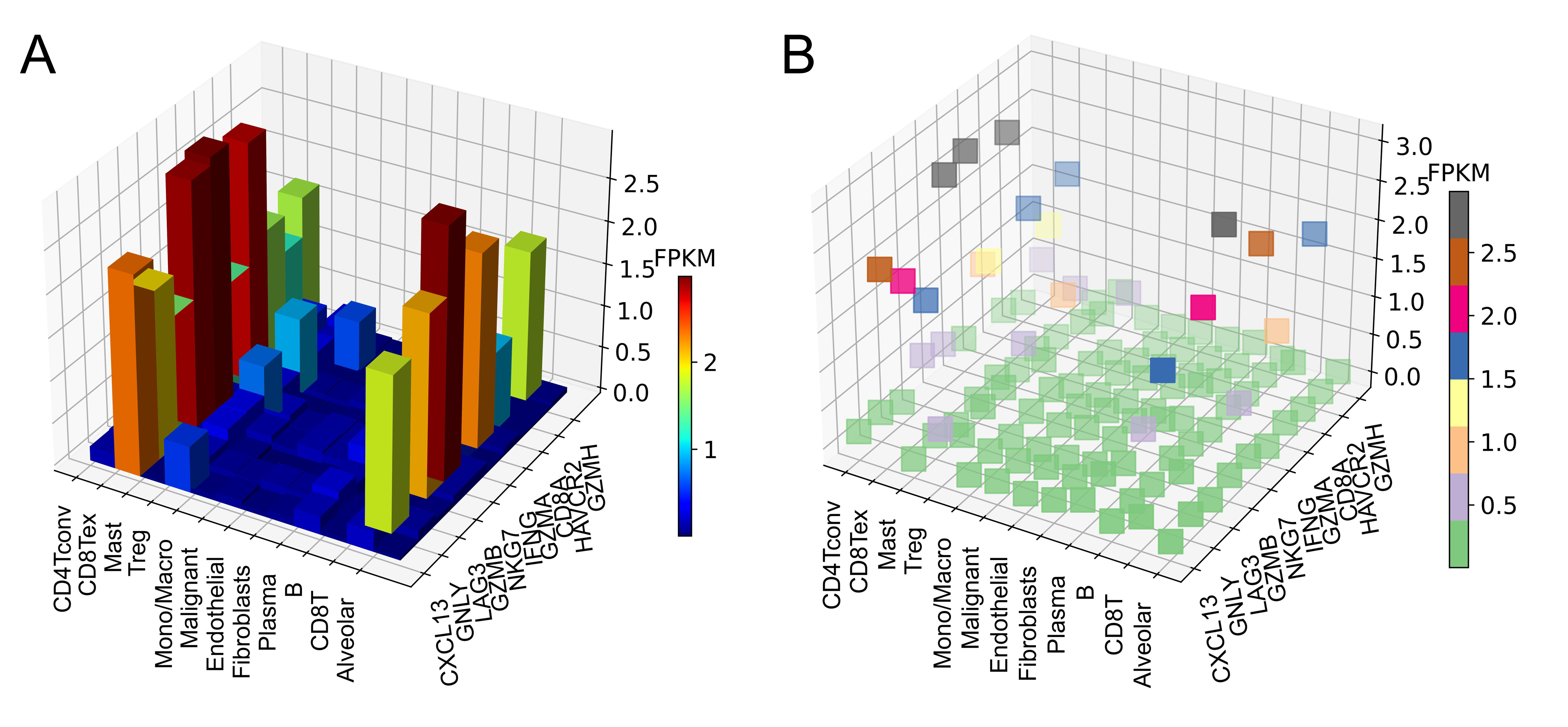
**

**Supplemental Figure 5. 3D plots produced by DataColor.** (A) 3D bar plot. (B) 3D scatter plot.

**
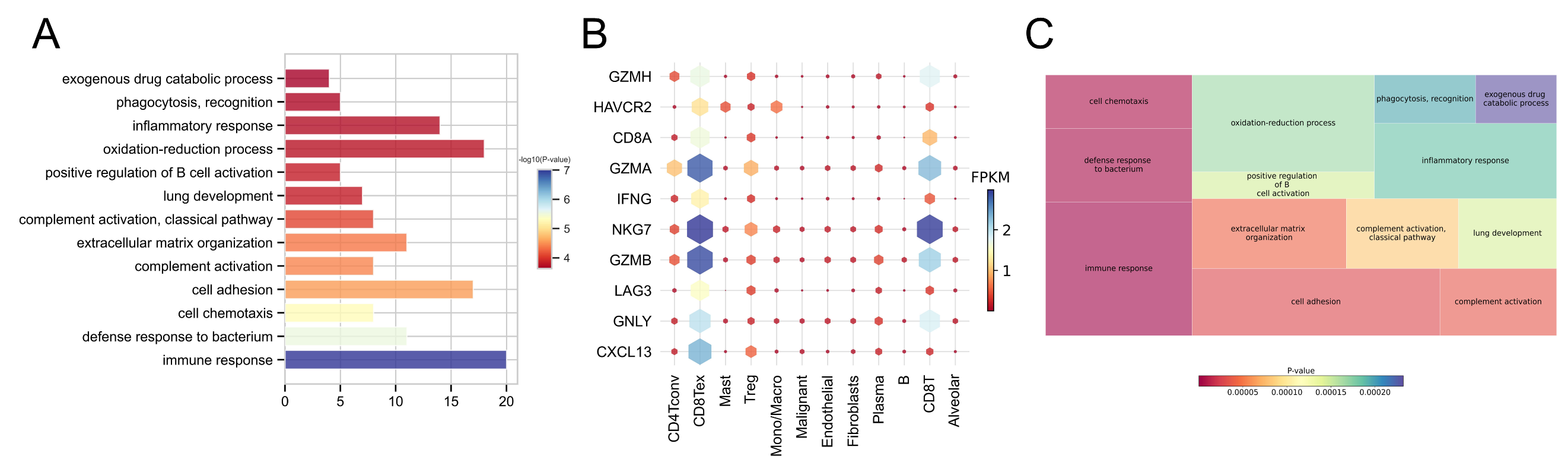
**

**Supplemental Figure 6. Figures drawn by Dot plot and Treemap produced by DataColor.** (A) Enrichment bar plot. (B) Dot plot. (C) Treemap plot.

**
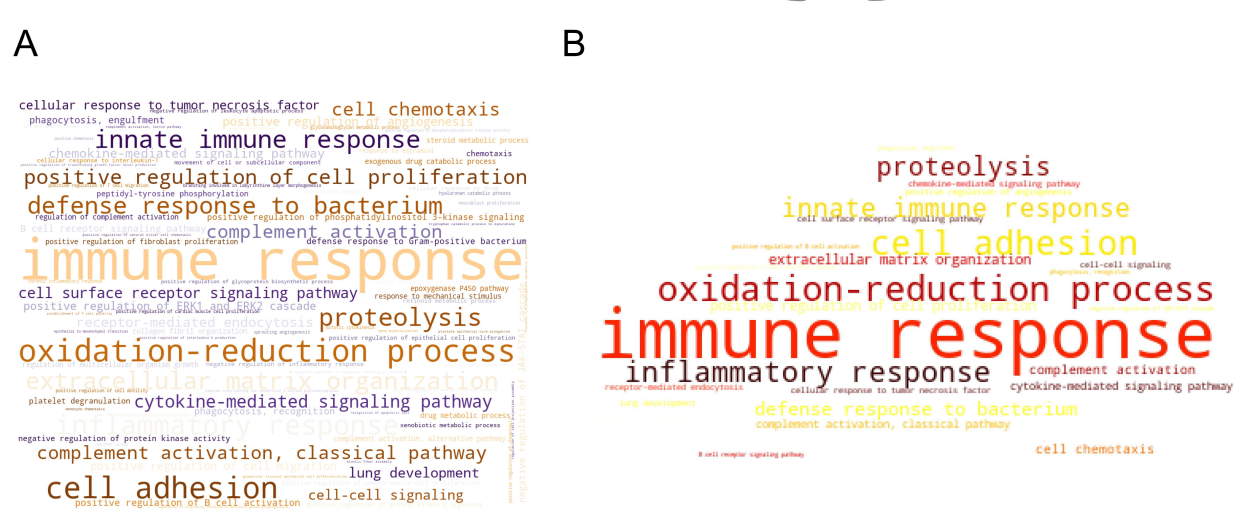
**

**Supplemental Figure 7. Wordcloud plots produced by DataColor.** (A) Wordcloud plot in square shape. (B) Wordcloud plot in car shape.
