# Supplementary material for: DataColor: unveiling biological data relationships through distinctive color mapping
Source: Hortic Res. 2023 Dec 21;11(2):uhad273. doi: 10.1093/hr/uhad273 (PMC10852383; doi:10.1093/hr/uhad273)
Supplement: Web_Material_uhad273 [file web_material_uhad273.zip › Supplementary file manual.docx]

#

# DataColor v1.0 Software Manual

[Sample 1](#_Toc15013)

[Overview 2](#_Toc13414)

[Download and install DataColor 2](#_Toc32073)

[Core programs 3](#_Toc2201)

[1. Sample collection 3](#_Toc23405)

[1.1 Calendar plotter 3](#_Toc20634)

[1.2 Correlation Matrix 5](#_Toc1094)

[1.3 Geographical map 7](#_Toc23320)

[1.4 Scatter and PCA plotter 9](#_Toc16086)

[1.5 Multivariable histogram 11](#_Toc25068)

[2 Structure and evolution 13](#_Toc14567)

[2.1 Hi-C plotter 13](#_Toc12719)

[2.3 Density peak plotter 17](#_Toc15508)

[2.4 Chr. linear plotter 19](#_Toc31294)

[2.5 Chr. circle plotter 21](#_Toc32157)

[3 Expression analysis 23](#_Toc13213)

[3.1 2D plotter 23](#_Toc12726)

[3.1.1 Heatmap 23](#_Toc30161)

[3.1.2.1 Circle heatmap 25](#_Toc21047)

[3.1.2.2 Circle heatmap (cluster) 27](#_Toc32213)

[3.2 3D plotter 29](#_Toc6325)

[3.2.1 3D heatmap 29](#_Toc30293)

[3.2.1.1 3D cluster by Row 31](#_Toc5643)

[3.2.1.2 3D cluster by Col 33](#_Toc2515)

[3.2.2 3D scatter plotter 34](#_Toc32686)

[3.2.3 3D Bar plotter 36](#_Toc3255)

[3.3 Isoheight plotter 37](#_Toc13125)

[3.3.1 Isoheight plotter 37](#_Toc19024)

[3.3.2 Isoheight plotter (Cluster) 39](#_Toc18945)

[4 Functional omics 41](#_Toc3494)

[4.1 Anatomogram plotter 41](#_Toc1790)

[4.2 Enrichment bar plotter 43](#_Toc26831)

[4.3 Bubble plotter 45](#_Toc36)

[4.4 Dot plotter 47](#_Toc19268)

[4.5 Network plotter 49](#_Toc5999)

[4.6 Treemap 51](#_Toc20247)

[4.7 Wordcloud plotter 53](#_Toc23073)

**Outputs**
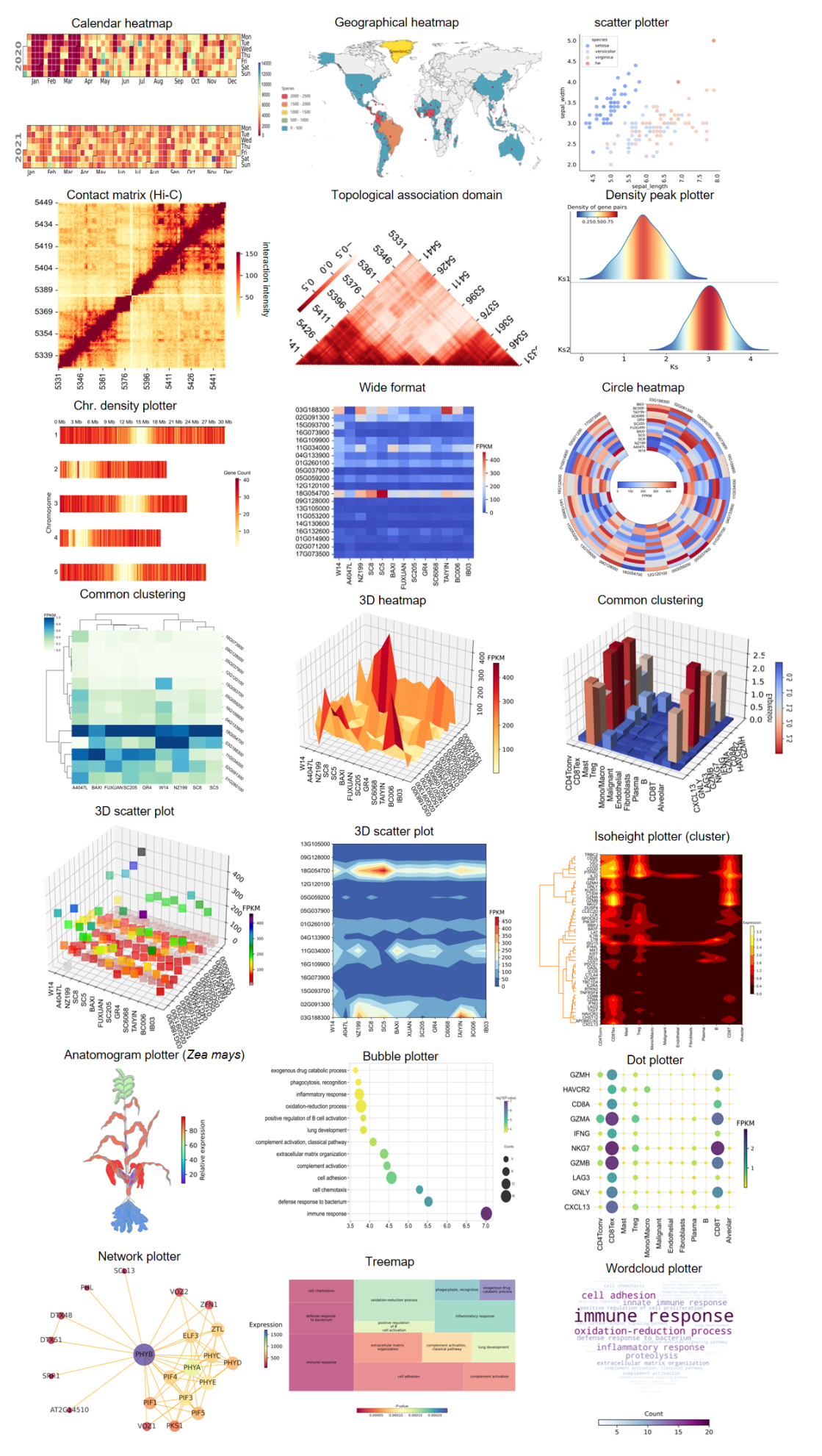


**Introductions**

With the rapid advancement of high-throughput histology technologies, the need for multidimensional and big data visualization is increasing along with the rapid increase of big biological data. Technological high speed innovation researchers have developed various bioinformatics software, software packages and other tools to draw visualization graphs, but also require users to have a basic knowledge of various programming languages. More and more users need comprehensive, fast, easy and intuitive retrieval of the information contained in big data. Easier visualization of large histological data to solve biological problems is extremely important.

To bridge this gap, we introduce DataColor—an all-encompassing software solution meticulously crafted to address this challenge. Our aim is to empower users with the ability to handle a wide array of data types through an assortment of tools, while simultaneously streamlining parameter selection for rapid insights and detailed enhancements. DataColor stands as a robust toolkit, encompassing 23 distinct tools. The defining characteristic of this toolkit is its adept utilization of the color spectrum, allowing for the representation of data spanning diverse types and magnitudes. Through the integration of advanced algorithms encompassing data clustering, normalization, squarified layouts, and customizable parameters, DataColor unveils an abundance of insights that lay hidden within the intricate relationships embedded in the data. Whether you find yourself navigating the analysis of expansive datasets or embarking on the quest to visualize intricate patterns, DataColor stands as the comprehensive and potent solution.

# Download and installation

1.Github download link

https://github.com/frankgenome/DataColor/releases

Link to manual and test data

https://github.com/frankgenome/DataColor

2.gitee download link

[https://gitee.com/heshuang-linda/DataColor](https://gitee.com/heshuang-linda/DataColor/releases/tag/Software)

3.figshare download link

https://figshare.com/account/home#/projects/169160

# Core programs

## Introduction to the input and output files

The input file is a csv file, which stores tabular data (numbers and text) in plain text, and is recommended to be opened using WORDPAD or Notepad, or by saving a new file and opening it in EXCEL. format files.

The output files are images, scalar images such as png and jpg, and svg vector images, and can also be produced as in pdf format.

# Sample collection

1.1 Calendar plotter

The Calendar plotter is an intuitive way to visualise data when doing feature analysis of time-series data.

**Select**

Menu -> Sample collection -> Calendar plotter

**Input**

Csv format or Excel format target series file


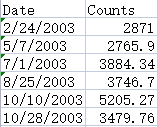


Fig: Input file.

**Output**

Generate image in the left canvas


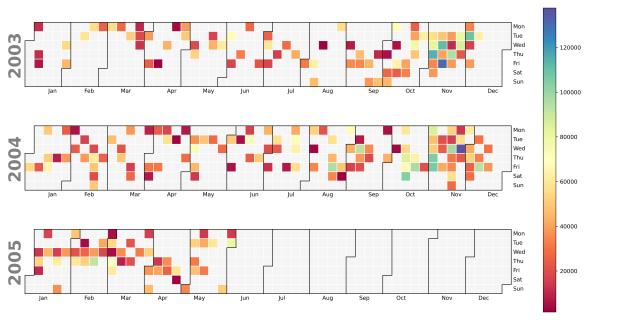


Fig: Calendar plotter output image

**Detailed instructions for use**

1. First of all, click the file button on the right, select the Csv format or Excel format file that needs to be visualized.

2. The default parameters have been set, if you need to adjust the parameters, please see the introduction below.

1. Show data displays the data values of each square on the heat map
2. The colour map contains 166 types and is divided into four main categories: Sequential colormaps: continuous colormaps, Diverging colormaps: discrete colormaps at both ends, Qualitative colormaps: discrete colormaps, Miscellaneous colormaps: Other colormaps. For example, 'Accent', 'Accent_r', 'Blues', 'Blues_r', 'BrBG', 'BrBG_r', etc. Each suffix _r colour map is a horizontal flip of the original colour bar.
3. Border widths are the dividing lines for each month of the Calendar plotter, if you need to adjust the colour, just select a value greater than 0 for the width.
4. 'Year lable colour' sets the colour of the month label.
5. Title can set the label.

3. After completing all configurations, click the 'Run' button to display or update the Treemap, which will appear in the canvas on the left.

4. Users can right-click the "Save" export option to export heat maps, and DataColor provides 900-1000dpi images for users to use. There are four image formats: png, jpg, pdf, and svg, to meet different needs.

## **1.2 Correlation Matrix**

Correlation Matrix is used to find the pairwise correlation of all columns in a data frame and to discover which variable is correlated with another variable by visualisation.

**Select**

Menu -> Expression analysis -> 2D plotter -> Correlation matrix

**Input**

Csv format or Excel format target series file


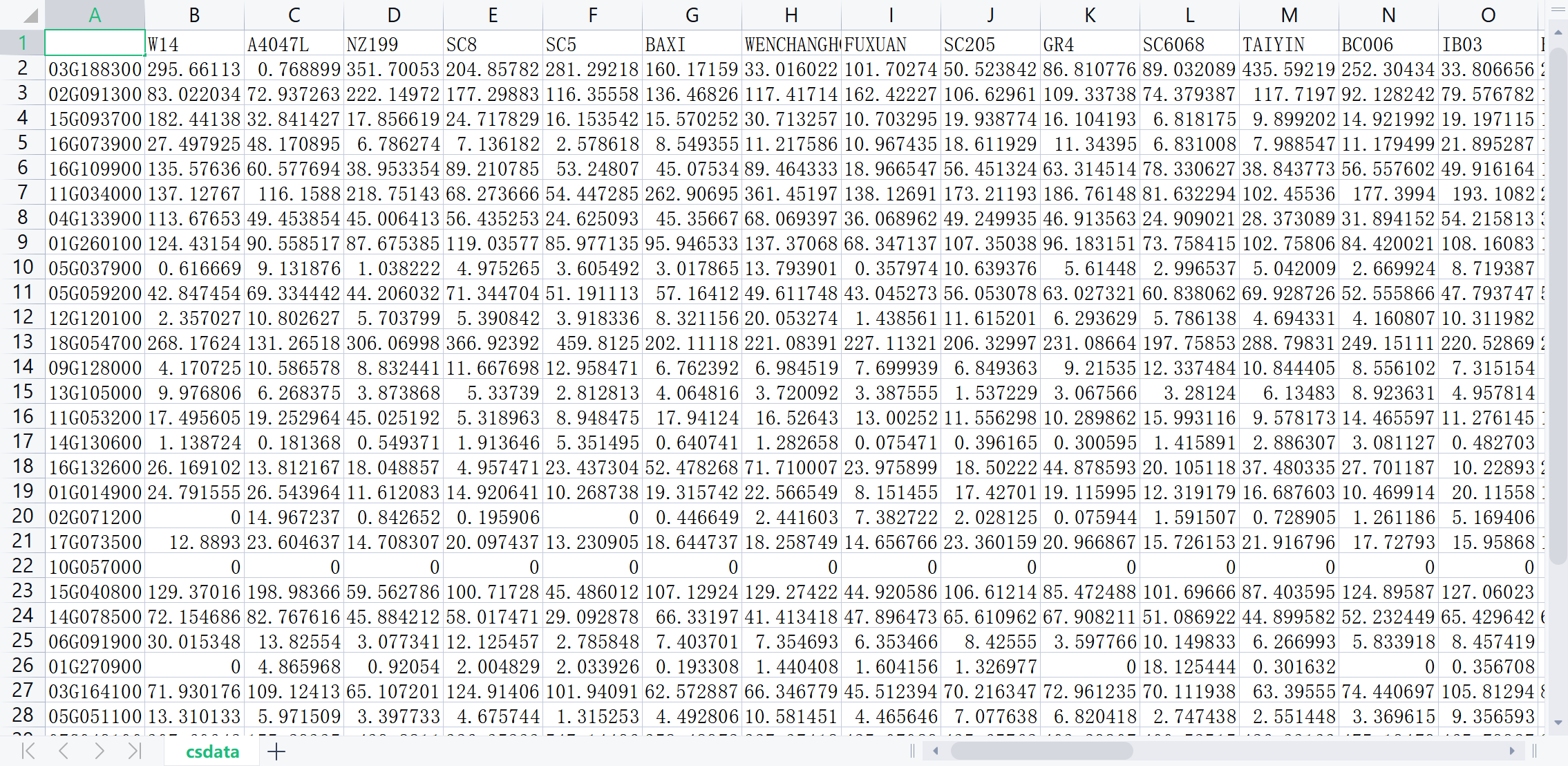


Fig:Input file.

**Output**

Generate image in the left canvas


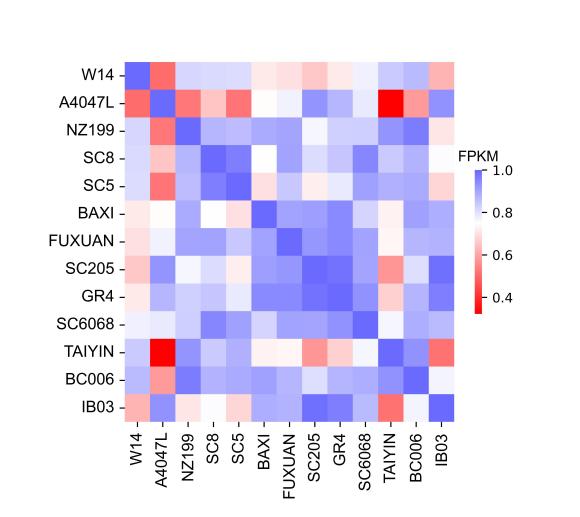


Fig: Correlation Matrix output image

**Detailed instructions for use**

1. First of all, click the file button on the right, select the Csv format or Excel format file that needs to be visualized.

2. The default parameters have been set, if you need to adjust the parameters, please see the introduction below

1. The Matrix type parameter can choose whether the generated image is a rectangle or a triangle.
2. Set the X-axis and Y-axis on and off, font, font size, color, rotation angle and step size.
3. Scale-min and Scale-max can customize the numerical range of the Color bar, or select None to be automatically defined by the system.
4. show data can display the data value of each square on the heat map, and can also adjust the display font size of the data value.
5. Scale label is divided into out, into, and in, and adjusts the orientation of the label teeth of the X and Y coordinate axes.
6. Cell square converts each small square generated in the heat map into a square.
7. The Color bar sets its direction, label font size, label name, size, and color.
8. Color map contains 166 types, mainly divided into the following four categories: Sequential colormaps: continuous colormaps, Diverging colormaps: divergent colormaps at both ends, Qualitative colormaps: discrete colormaps, Miscellaneous colormaps: other colormaps. Such as 'Accent', 'Accent_r', 'Blues', 'Blues_r', 'BrBG', 'BrBG_r', etc. Each suffix _r color map is the original color bar flipped horizontally.
9. Line widths are the dividing lines of the heat map. If necessary, you can adjust the color and select a value greater than 0 for the width.

3. Once all configurations are complete, click on the "Run" button to display or update the heat map, which will appear in the canvas on the left.

4. You can export the plotter by right clicking on the 'Save' export option. Four image formats are available: png, jpg, pdf and svg to suit different needs.

## **1.3 Geographical map**

Geographical plotter are map-like heat maps that can be displayed in a visual way with colour values based on the values of each geographical location in the data.

**Select**

Menu -> Sample collection -> Geographical map

**Input**

Csv format or Excel format target series file


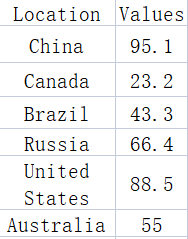

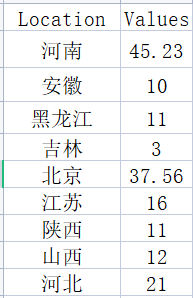


Fig: Input file.

**Output**

Generate image in the left canvas


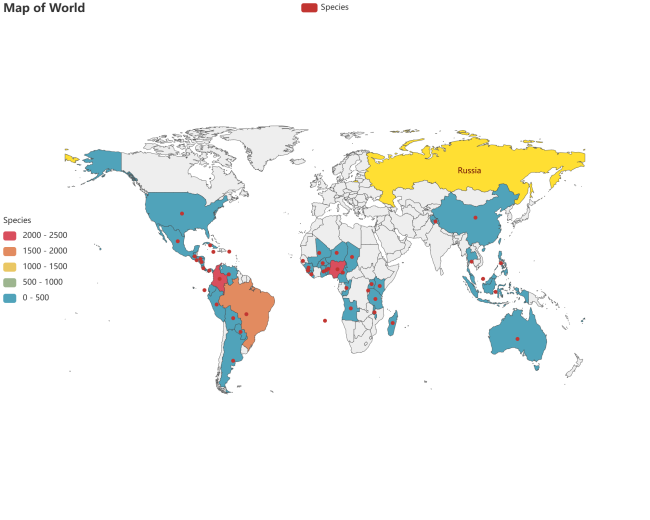


Fig: Geographical map output image

**Detailed instructions for use**

1. First of all, click the file button on the right, select the Csv format or Excel format file that needs to be visualized.

2. The default parameters have been set, if you need to adjust the parameters, please see the introduction below

1. Type input the map type, such as "world", "china", "贵州", etc.
2. Width and Height set the width and height of the map.
3. Scale-min and Scale-max to customise the total range of values for the Color bar.
4. Color selects whether the map has a colour bar or not.
5. Color bar sets its orientation selection.
6. Title, table, Color bar can set the label name.

3.After completing all the configurations, click on the 'Run' button to display or update the Map, which will pop up as a web page where the user can click on the download button to save the image.

**1.4 Scatter and PCA plotter**

Datacolor adds the Scatter plot function, which refers to the distribution of data points on the plane of the Cartesian coordinate system. 23 different shapes such as point, diamond, circle, and star can be used to draw the graph. In addition to the color representing the data, the size of the point and the depth of the color are consistent, and at the same time represent the size of the value. And it can realize point synchronous enlargement and synchronous reduction.

PCA (Principal Component Analysis), the principal component analysis method, is the most widely used data dimensionality reduction algorithm. The main idea of PCA is to map n-dimensional features to k-dimensional features. This k-dimensional feature is a new orthogonal feature, also known as the principal component. It is a k-dimensional feature reconstructed based on the original n-dimensional feature.

**Select**

Menu -> Sample collection -> Scatter and PCA plotter

**Input**

Csv format or Excel format target series file


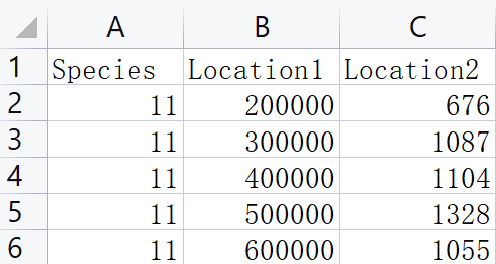


Fig: Input file.

**Output**

Generate image in the left canvas


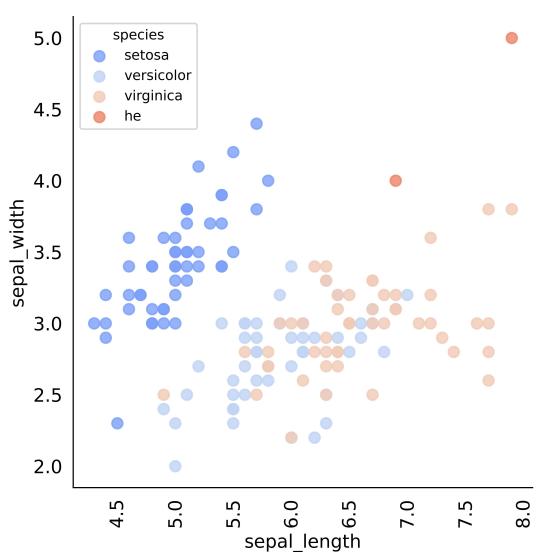


Fig: Scatter and PCA plotter output image

**Detailed instructions for use**

1. First of all, click the file button on the right, select the Csv format or Excel format file that needs to be visualized.

2. The default parameters have been set, if you need to adjust the parameters, please see the introduction below.

1. The PCA parameter is used to select whether the data is subjected to PCA calculation, and the Number of feature dimensions parameter is used to select the number of feature dimensions for data reduction.
2. Set the X-axis and Y-axis on and off, font size, color, rotation angle and step size
3. Color bar sets its direction, label font size, label name, size, color, position, and font size.
4. The color map contains 166 types, which are mainly divided into the following four categories: Sequential colormaps: continuous color map, Diverging colormaps: color map diverging at both ends, Qualitative colormaps: discretized color map, Miscellaneous colormaps: other color maps. Such as 'Accent', 'Accent_r', 'Blues', 'Blues_r', 'BrBG', 'BrBG_r', etc., each suffix _r color map is the original color bar flipped horizontally.
5. Dot shape sets 23 dot shapes
6. Dot size magnification can adjust the size of the point
7. The Transparency parameter can adjust the transparency of the point color

3. After completing all configurations, click the 'Run' button to display or update the Treemap, which will appear in the canvas on the left.

4. Users can right-click the "Save" export option to export heat maps, and DataColor provides 900-1000dpi images for users to use. There are four image formats: png, jpg, pdf, and svg, to meet different needs.

**1.5 Multivariable histogram**

Multivariable histogram is usually used in biology to plot data of multiple variables or characteristics to study the relationships and distributions between them.

**Select**

Menu -> Sample collection -> Multivariable histogram

**Input**

Csv format or Excel format target series file


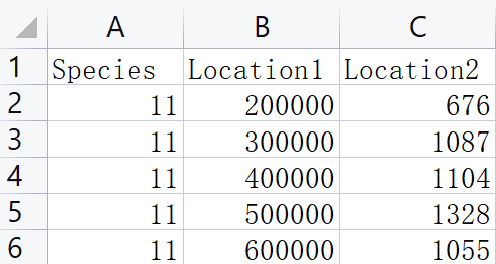


Fig: Input file.

**Output**

Generate image in the left canvas


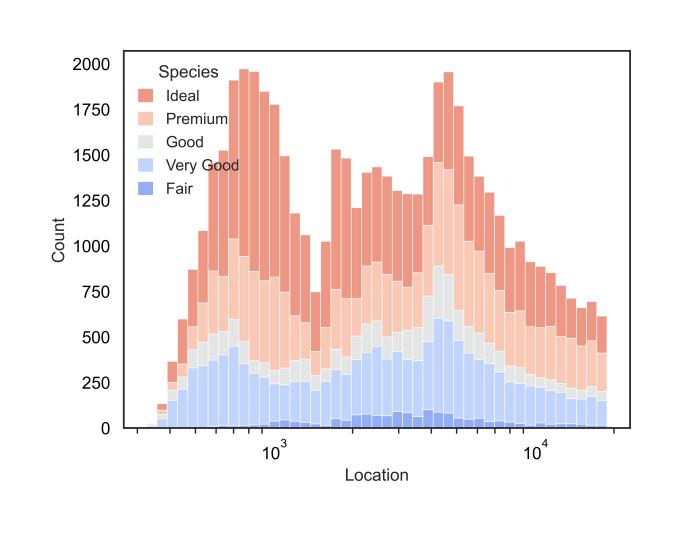


Fig: Multivariable histogram output image

**Detailed instructions for use**

1. First of all, click the file button on the right, select the Csv format or Excel format file that needs to be visualized.

2. The default parameters have been set, if you need to adjust the parameters, please see the introduction below.

1. Set the X-axis and Y-axis on and off, font size, color, rotation angle and step size
2. Color bar sets its direction, label font size, label name, size, color, position, and font size.
3. The color map contains 166 types, which are mainly divided into the following four categories: Sequential colormaps: continuous color map, Diverging colormaps: color map diverging at both ends, Qualitative colormaps: discretized color map, Miscellaneous colormaps: other color maps. Such as 'Accent', 'Accent_r', 'Blues', 'Blues_r', 'BrBG', 'BrBG_r', etc., each suffix _r color map is the original color bar flipped horizontally.

3. After completing all configurations, click the 'Run' button to display or update the Treemap, which will appear in the canvas on the left.

4. Users can right-click the "Save" export option to export heat maps, and DataColor provides 900-1000dpi images for users to use. There are four image formats: png, jpg, pdf, and svg, to meet different needs.

# **2 Structure and evolution**

## **2.1 Hi-C plotter**

High-throughput chromosome conformation capture is an experimental technique for studying genome-wide three-dimensional conformations and analysing chromatin fragment interactions.Hi-C aims to understand the three-dimensional conformation of chromatin in the nucleus, to obtain chromatin fragments in close spatial proximity or with interactions in the nucleus and to better study chromatin interactions within or between chromatin. interactions and genome-wide regulation of gene regulatory elements.If you are hic format files, before using this function you need to use HiCexplorer software hicConvertFormat to convert hic/ hicpro matrix files to ginteractions format, and then to txt or csv three column format files.

**Select**

Menu -> Structure and evolution -> Hi-C contact matrix

**Input**

TxT format target series file


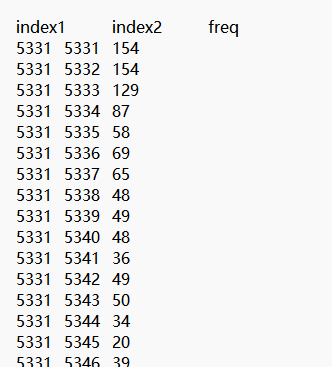


Fig: txt formatted file.

**Output**

Generate image in the left canvas


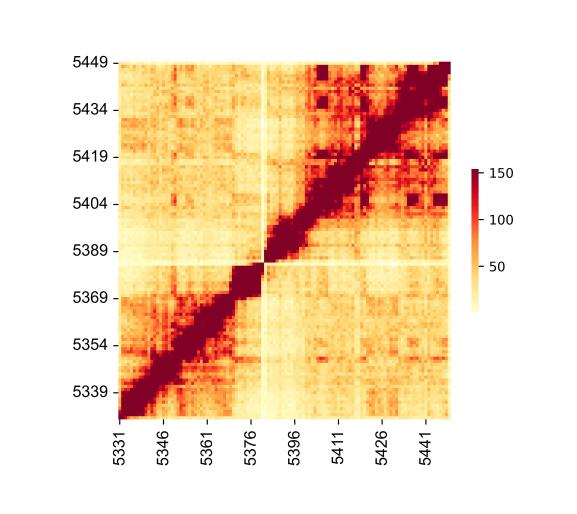


Fig: Hi-C contact matrix output image

**Detailed instructions for use**

1. First click on the file button on the right and select txt format file you need to visualize.

## Default parameters have been set, if you need to adjust the parameters, please see the following introduction

## Set the X-axis and Y-axis on/off, font, font size, color, rotation angle and step length.

## Scale-min and Scale-max can customize the value range of Color bar, or you can choose None, which is automatically defined by the system.

## show data can display the data value of each square on the heat map, and also can adjust the display font size of the data value.

## Scale lable is divided into out, into, and in, which adjusts the orientation of the label teeth of X and Y coordinate axes.

## Cell square is to turn every small square generated by the heat map into a square.

## Color bar sets its direction, label font size, label name, size, and color.

## Color map contains 166 kinds, mainly divided into the following four categories: Sequential colormaps: continuous color maps, Diverging colormaps: color maps with dispersion at both ends, Qualitative colormaps: discrete color maps, Miscellaneous colormaps: Other colormaps. Such as 'Accent', 'Accent_r', 'Blues', 'Blues_r', 'BrBG', 'BrBG_r', etc. Each suffix _r color map is the original color bar horizontally flipped.

## Line widths is the split line of the heat map, if you need to be able to adjust the color, the width will be greater than 0 value can be selected.

3. Once all configurations are complete, click on the "Run" button to display or update the heat map, which will appear in the canvas on the left.

4. You can export the plotter by right clicking on the 'Save' export option. Four image formats are available: png, jpg, pdf and svg to suit different needs.

## **2.2 Topologically associated domain**

Genome interoperation mapping is essentially a symmetric matrix with equal information on both sides of the diagonal. The intensity of the interactions ranges from weak to strong, with the colour of the cells transitioning from white to red. There are small triangular regions repeated out of the bottom edge, almost all of which are red inside, indicating a high frequency of interactions between chromatin segments within these regions, such regions are called self-interaction regions, while the frequency of interactions between adjacent triangular regions is low, with red triangular regions corresponding to interactions between regions within TADs, and black regions corresponding to inter-TAD The red triangular areas correspond to the interactions within the TAD, while the black areas correspond to the interactions between TADs. The red triangular region corresponds to the interactions of the intra-TAD region, while the black region corresponds to the interactions between TADs. The triangular interactions are presented on the triangular interactions map, which corresponds to a number of small red triangles on the bottom edge, while the triangles correspond to interactions that are all white. format file, you will need to use HiCexplorer's hicConvertFormat to convert the .hic/ hicpro matrix file to ginteractions format, and then to a three-column file in txt or csv before using this function.

**Select**

Menu -> Structure and evolution -> Topologically associated domain

**Input**

TxT format target series file


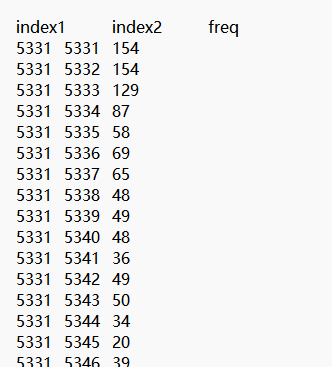


Fig: Input file.

**Output**

Generate image in the left canvas


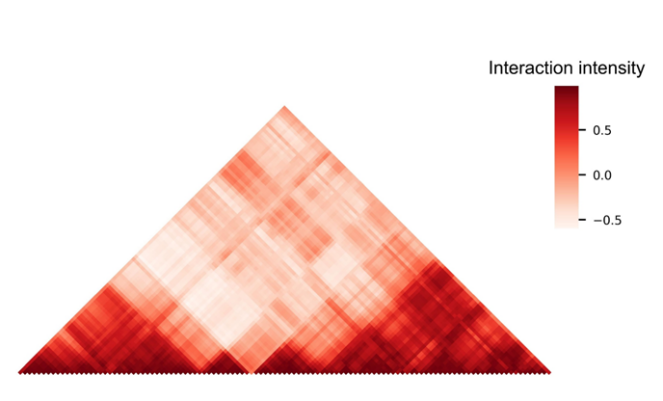


Fig: Topologically associated domain output image

**Detailed instructions for use**

1. First click on the file button on the right and select txt format file you need to visualize.

## Default parameters have been set, if you need to adjust the parameters, please see the following introduction

## Set the X-axis and Y-axis on/off, font, font size, color, rotation angle and step length.

## Scale-min and Scale-max can customize the value range of Color bar, or you can choose None, which is automatically defined by the system.

## show data can display the data value of each square on the heat map, and also can adjust the display font size of the data value.

## Scale lable is divided into out, into, and in, which adjusts the orientation of the label teeth of X and Y coordinate axes.

## Cell square is to turn every small square generated by the heat map into a square.

## Color bar sets its direction, label font size, label name, size, and color.

## Color map contains 166 kinds, mainly divided into the following four categories: Sequential colormaps: continuous color maps, Diverging colormaps: color maps with dispersion at both ends, Qualitative colormaps: discrete color maps, Miscellaneous colormaps: Other colormaps. Such as 'Accent', 'Accent_r', 'Blues', 'Blues_r', 'BrBG', 'BrBG_r', etc. Each suffix _r color map is the original color bar horizontally flipped.

## Line widths is the split line of the heat map, if you need to be able to adjust the color, the width will be greater than 0 value can be selected.

3. Once all configurations are complete, click on the "Run" button to display or update the heat map, which will appear in the canvas on the left.

4. You can export the plotter by right clicking on the 'Save' export option. Four image formats are available: png, jpg, pdf and svg to suit different needs.

2.3 Density peak plotter

Density peak plot mainly shows the same X-axis data, which can be time series, genetic data, etc., corresponding to different Y-axis data, and clearly shows the relationship between different data and variables.

**Select**

Menu -> Structure and evolution -> Density peak plotter

**Input**

Csv format or Excel format target series file


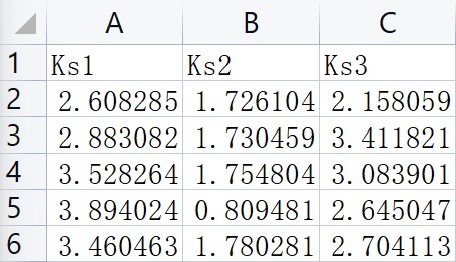


Fig: Input file.

**Output**

Generate image in the left canvas


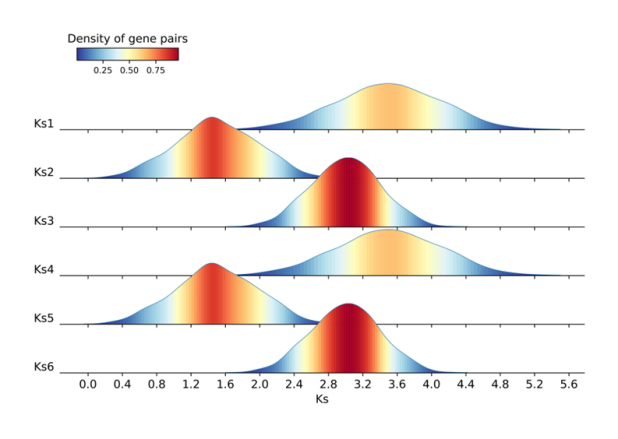


Fig: Density peak plotter output image

**Detailed instructions for use**

1. First, click the file button on the right and select the Csv or Excel file that you want to visualize

2. The default parameters have been set. If you need to adjust the parameters, please see the following

Detailed usage:

1. Set X axis and Y axis on and off, font, size, color, rotation Angle.
2. The X-axis label number parameter is used to set the number of x-axis labels.
3. Color bar sets its direction, label size, label name, size, color, position, and size.
4. color map contains 166 kinds, mainly divided into the following four categories: Sequential colormaps: continuous colormap, Diverging colormaps: divergent colormap at both ends, Qualitative colormaps: Miscellaneous colormaps: miscellaneous colormaps. For example, 'Accent', 'Accent_r', 'Blues', 'Blues_r', 'BrBG', 'BrBG_r', etc., each suffix _r color map is the original color bar flipped horizontally.
5. Vertical spacing Sets the distance between subplots
6. Y-axis lable sets the rotation direction and font size
7. X-axis lable sets the input word and size

3. After completing all configurations, click the "Run" button to display or update the heat map. The heat map will appear in the left canvas.

4. You can right-click Save to export the heat map. DataColor provides six data ranges from 300 dpi to 1000dpi. There are four image formats: png,.jpg, pdf,.SVG to meet different needs.

2.4 Chr. linear plotter

The Chromosome Density tool is a mapping tool for representing protein-protein interactions, gene regulatory networks, and metabolic pathways. This facilitates the study of functional associations between biomolecules, regulation of biological pathways, and mechanisms of disease pathogenesis.

**Select**

Menu -> Structure and evolution -> Chr. linear plotter

**Input**

Csv format or Excel format target series file


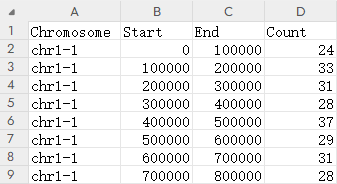

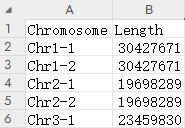


Fig: Input file.

**Output**

Generate image in the left canvas


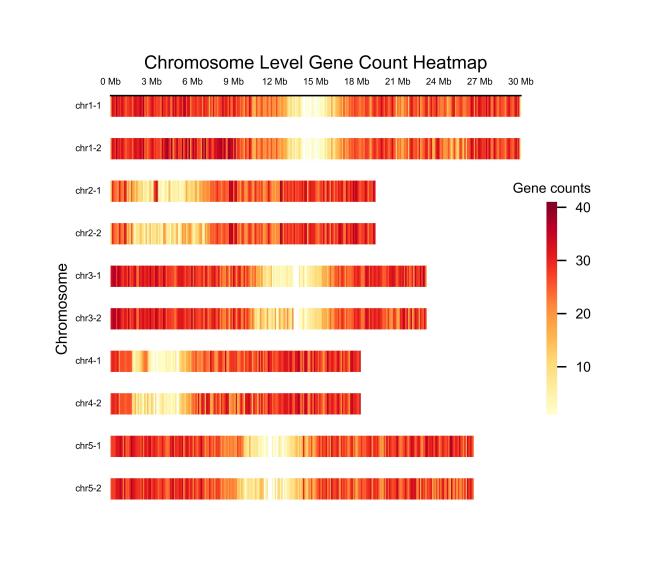


Fig: Chr. linear plotter output image

**Detailed instructions for use**

1. First, click the file button on the right and select the Csv or Excel file that you want to visualize

2. The default parameters have been set. If you need to adjust the parameters, please see the following

Detailed usage:

1. Set the X-axis and Y-axis on and off, font, font size, color, rotation angle and step size
2. Scale-min and Scale-max can customize the value range of Color bar, or select None, which will be automatically defined by the system.
3. Scale lable is divided into out, into, and in, and adjusts the orientation of the label teeth of the X and Y coordinate axes.
4. Color bar sets its direction, label font size, label name, size and color.
5. The color map contains 166 types, which are mainly divided into the following four categories: Sequential colormaps: continuous color map, Diverging colormaps: color map diverging at both ends, Qualitative colormaps: discretized color map, Miscellaneous colormaps: other color maps. Such as 'Accent', 'Accent_r', 'Blues', 'Blues_r', 'BrBG', 'BrBG_r', etc., each suffix _r color map is the original color bar flipped horizontally.
6. Title and Y-axis lable set the input text

3. After completing all configurations, click the "Run" button to display or update the heat map. The heat map will appear in the left canvas.

4. You can right-click Save to export the heat map. DataColor provides six data ranges from 300 dpi to 1000dpi. There are four image formats: png,.jpg, pdf,.SVG to meet different needs.

2.5 Chr. circle plotter

Chr. circle plotter can be used for many types of data circles, including heat maps, column charts, line charts, and scatter plots. Suitable for gene expression data, proteomic data, genomic data, chromatin interaction data, DNA methylation data, ChIP-seq data, transcriptome data, epigenomic data, mutation and structural variation data.

**Select**

Menu -> Structure and evolution -> Chr. circle plotter

**Input**

Csv format target series file


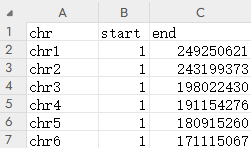

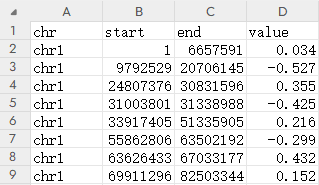


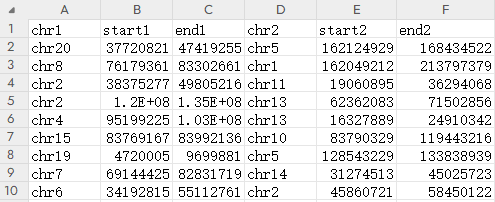


Fig: Input file.

**Output**

Generate image in the left canvas


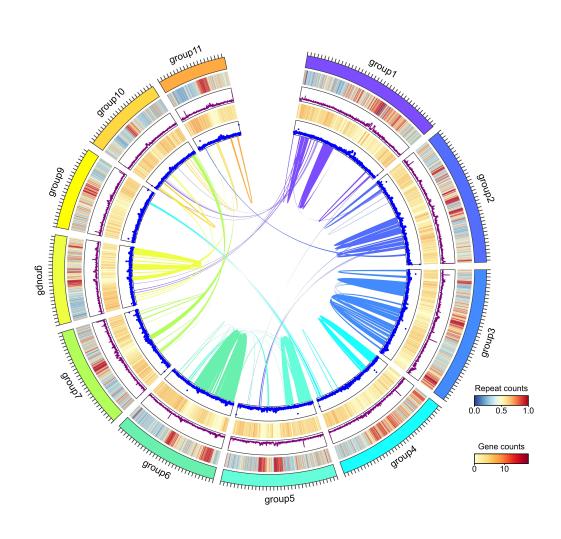


Fig: Chr. circle plotter output image

**Detailed instructions for use**

1. First, click the file button on the right and select the Csv file that you want to visualize

2. The default parameters have been set. If you need to adjust the parameters, please see the following

Detailed usage:

1. Enter the drawing file in Select file, Select file on length to enter the chromosome length file, Select file on circle1-4 to enter the data for each circle, Select file on Links, and enter the chromosome collinearity data.
2. Set the Tick on and off and font size
3. Color map 1-4 contains 166 types, mainly divided into the following four categories: Sequential colormaps: continuous colormaps, Diverging colormaps: color maps that diverge at both ends, Qualitative colormaps: discrete colormaps, Miscellaneous colormaps: other colors picture. Such as 'Accent', 'Accent_r', 'Blues', 'Blues_r', 'BrBG', 'BrBG_r', etc. Each suffix _r color map is the original color bar flipped horizontally.
4. Color bar label 1-4 correspond to the Color bar labels of the four circles respectively.
5. Color bar sets the label font size and scale font size.
6. The Scatter type ‘Shape’ sets 15 point shapes, and the ‘Color’ parameter sets the color.
7. The Bar type sets the ‘Color’ parameter to set the color, and the ‘Line style’ parameter sets the 4 line types.
8. Line type sets parameters for color and line width

3. After completing all configurations, click the "Run" button to display or update the heat map. The heat map will appear in the left canvas.

4. You can right-click Save to export the heat map. DataColor provides six data ranges from 300 dpi to 1000dpi. There are four image formats: png,.jpg, pdf,.SVG to meet different needs.

3 Expression analysis

3.1 2D plotter

**3.1.1 Heatmap**

The "Heatmap" tool generates user-friendly heat maps, applicable in scenarios where clustering isn't required but a direct representation of the heatmap is needed, as well as in clustered heatmap scenarios. This tool incorporates standardization parameters, divided into standard and Z-score options, encompassing both column and row standardization to alleviate differences in data magnitude. Clustering algorithms are primarily utilized in unsupervised learning and structured data analysis.

**Select**

Menu -> Expression analysis -> 2D plotter -> Heatmap

**Input**

Csv format or Excel format target series file


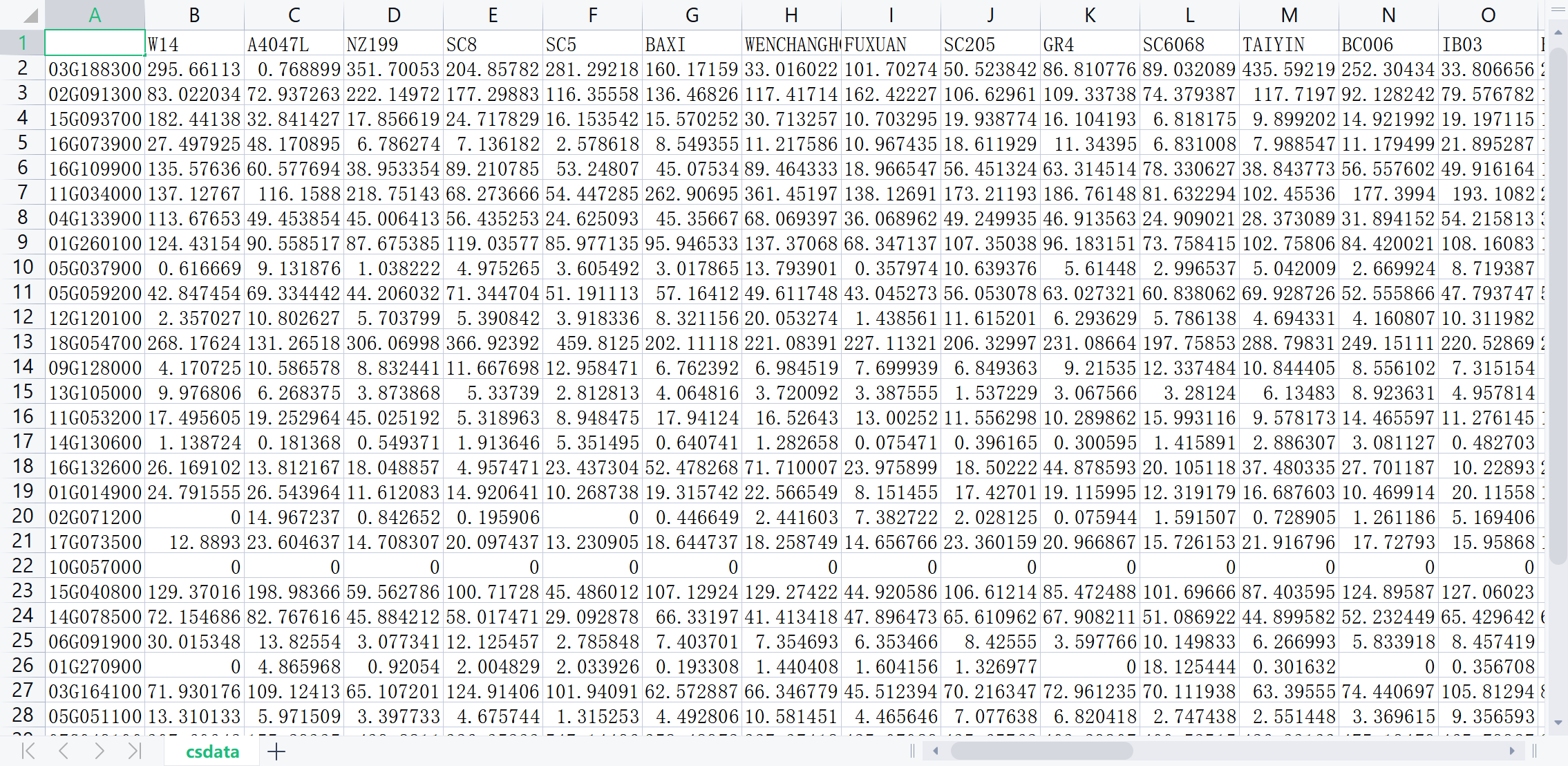


Fig: Input file.

**Output**

Generate image in the left canvas


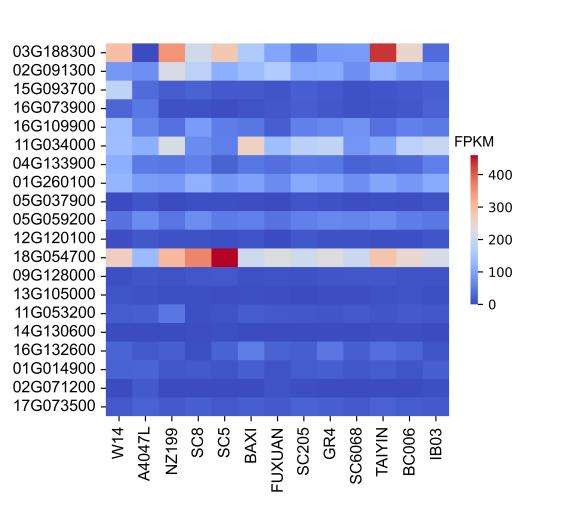

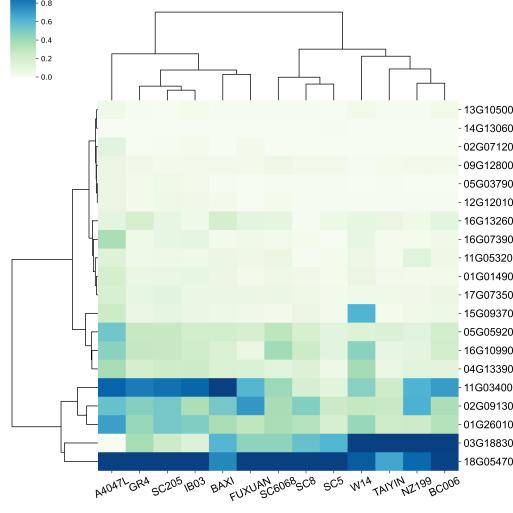


Fig: Heatmap output image

**Detailed instructions for use**

1. First of all, click the file button on the right, select the Csv format or Excel format file that needs to be visualized.
2. Default parameters have been set, if you need to adjust the parameters please see the following introduction.
3. X-axis and Y-axis set the X-axis and Y-axis on/off, font, font size, colour, rotation angle and step length.
4. Row cluster and Col cluster control the clustering of rows and columns on and off.
5. The Normalization parameter standardizes the heat map. You can choose Standard and Z-score two processing methods, and choose row processing or column processing.
6. Scale-min and Scale-max allow you to define the range of values for the Color bar, or you can choose None, which is automatically defined by the system.
7. show data allows you to display the data values of each square on the heat map, and to adjust the font size of the data values.
8. Metric allows you to choose from 7 clustering methods.
9. Method allows you to choose from 22 distance measures.
10. Cell square converts each of the resulting small squares of the heat map into a square.
11. Color bar sets its orientation, label font size, label name, size and colour.
12. The colour map contains 166 types and is divided into the following four main categories: Sequential colormaps: continuous colormaps, Diverging colormaps: colormaps with dispersion at both ends, Qualitative colormaps: discrete colormaps, Miscellaneous colormaps. Other colormaps. For example, 'Accent', 'Accent_r', 'Blues', 'Blues_r', 'BrBG', 'BrBG_r', etc. Each suffix _r colour map is a horizontal flip of the original colour bar.
13. Line widths are the dividing lines of the heat map, if you need to adjust the colour, select a value greater than 0 for the width.
14. Column comment enables column commenting, comment column name enters the name of the column to comment on, which is also the name of the comment column in the diagram.
15. Classify color1, Classify color2, Classify color3 select what colour.
16. Tree style sets the shape, width and colour of the clustering tree.
17. Robust opens the possibility to use quantile to calculate the colour mapping range.

3. Once all configurations are complete, click the "Run" button to display or update the heat map, which will appear in the canvas on the left.

4. The user can export the plotter by right clicking on the 'Save' export option, DataColor offers a range of images from 900-1000 dpi for use. Four image formats are available: png, jpg, pdf and svg to suit different needs.

3.1.2.1 Circle heatmap

Circle plotter, as the name suggests, is a form of heat map. The advantage of Circle plotter is that it can display multiple aspects on a single graph, which is suitable for the study of multiple groups or multiple omics, and can reveal the change rules and connections of different omics.

**Select**

Menu -> Expression analysis -> 2D plotter -> Circle heatmap

**Input**

Csv format or Excel format target series file


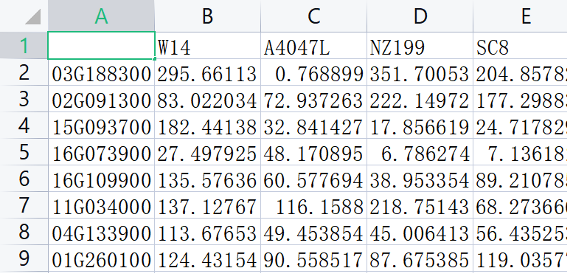


Fig:Input file.

**Output**

Generate image in the left canvas


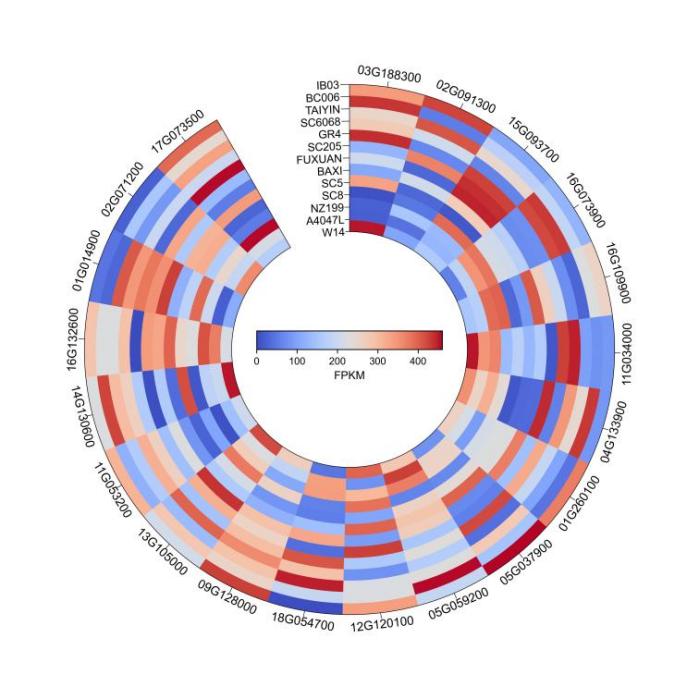


Fig: Circle heatmap output image

**Detailed instructions for use**

1. First, click the file button on the right and select the Csv or Excel file that you want to visualize

2. The default parameters have been set. If you need to adjust the parameters, please see the following description

1. The Cluster by parameter is a clickable button, which will jump to the corresponding clustering tool.
2. Set X axis and Y axis on and off, font, size, color, rotation Angle.
3. show data can display the data value of each square on the heat map, and can also adjust the display size of the data value.
4. Color bar sets its direction, label size, label name, height and width, and color.
5. color map contains 166 kinds, mainly divided into the following four categories: Sequential colormaps: continuous colormap, Diverging colormaps: divergent colormap at both ends, Qualitative colormaps: Miscellaneous colormaps: miscellaneous colormaps. For example, 'Accent', 'Accent_r', 'Blues', 'Blues_r', 'BrBG', 'BrBG_r', etc., each suffix _r color map is the original color bar flipped horizontally.
6. Border widths are the dividing lines of the heat map. The color can be adjusted if necessary, and the width can be selected as a value greater than 0.

3. After completing all configurations, click the "Run" button to display or update the heat map. The heat map will appear in the left canvas.

## 4. You can right-click Save to export the heat map. DataColor provides six data ranges from 300 dpi to 1000dpi. There are four image formats: png,.jpg, pdf,.SVG to meet different needs.

3.1.2.2 Circle heatmap (cluster)

Circle heatmap (cluster) is a heatmap pattern that combines a circle heatmap with tree clustering.

**Select**

Menu -> Expression analysis -> 2D plotter -> Circle heatmap (cluster)

**Input**

Csv format or Excel format target series file


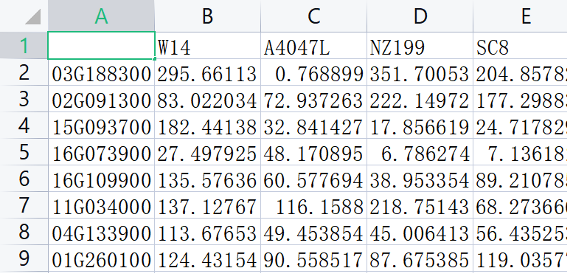


Fig:Input file.

**Output**

Generate image in the left canvas


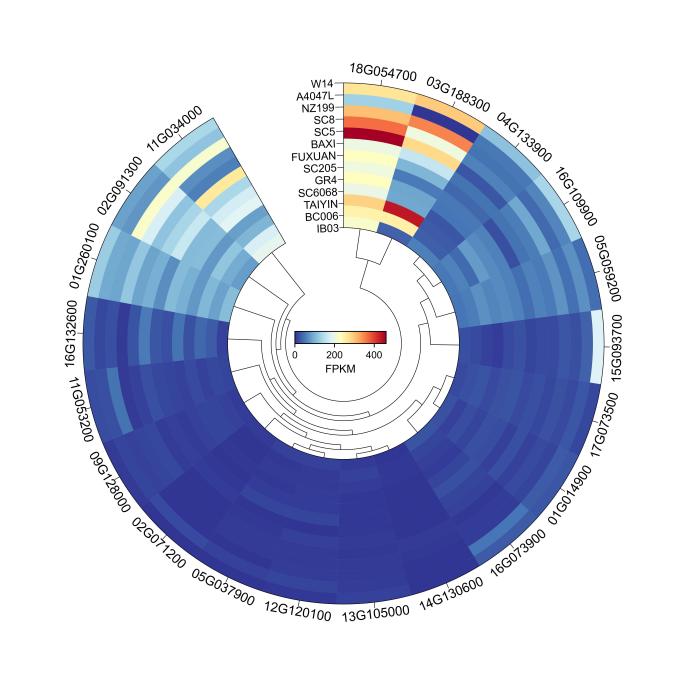


Fig: Circle heatmap (cluster) output image

**Detailed instructions for use**

1. First, click the file button on the right and select the Csv or Excel file that you want to visualize

2. The default parameters have been set. If you need to adjust the parameters, please see the following description

1. Metric can choose from 7 clustering methods
2. Method can choose from 22 distance measures
3. Set X axis and Y axis on and off, font, size, color, rotation Angle.
4. show data can display the data value of each square on the heat map, and can also adjust the display size of the data value.
5. Color bar sets its direction, label size, label name, height and width, and color.
6. color map contains 166 kinds, mainly divided into the following four categories: Sequential colormaps: continuous colormap, Diverging colormaps: divergent colormap at both ends, Qualitative colormaps: Miscellaneous colormaps: miscellaneous colormaps. For example, 'Accent', 'Accent_r', 'Blues', 'Blues_r', 'BrBG', 'BrBG_r', etc., each suffix _r color map is the original color bar flipped horizontally.
7. Border widths are the dividing lines of the heat map. The color can be adjusted if necessary, and the width can be selected as a value greater than 0.

3. After completing all configurations, click the "Run" button to display or update the heat map. The heat map will appear in the left canvas.

## 4. You can right-click Save to export the heat map. DataColor provides six data ranges from 300 dpi to 1000dpi. There are four image formats: png,.jpg, pdf,.SVG to meet different needs.

3.2 3D plotter

3.2.1 3D heatmap

The 3D plotter displays three dimensions of data at the same time, making the results of complex data analysis much clearer and easier to obtain directly from the graph.

**Select**

Menu -> Expression analysis -> 3D plotter -> 3D heatmap

**Input**

Csv format or Excel format target series file


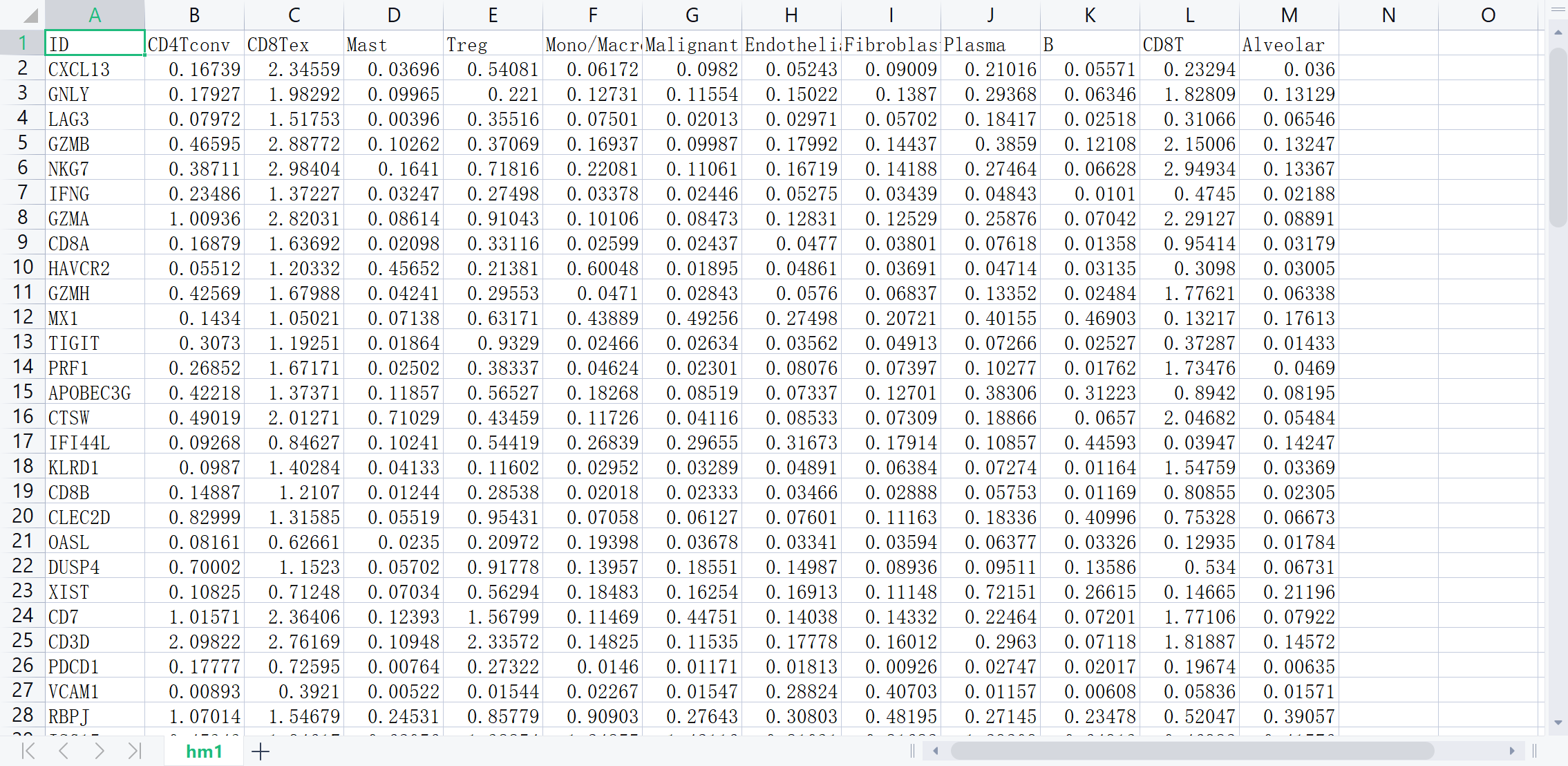


Fig: Input file.

**Output**

Generate image in the left canvas


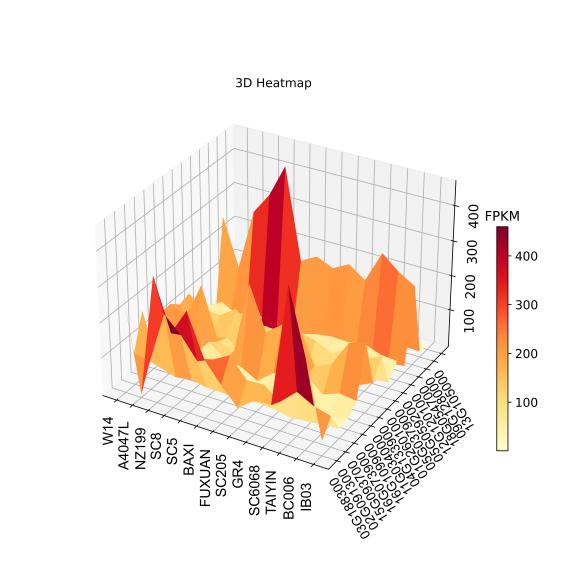


Fig: 3D plotter(Non-cluster) output image

**Detailed instructions for use**

1. First of all, click the file button on the right, select the Csv format or Excel format file that needs to be visualized.

2. The default parameters have been set, if you want to adjust the parameters please see the following introduction.

1. X-axis, Y-axis, Z-axis set the X-axis, Y-axis, Z-axis on and off, font, font size, colour, rotation angle.
2. The Cluster by parameter is a clickable button. You can select row clustering and column clustering, and it will jump to the corresponding clustering tool.
3. Color bar sets the direction, label size, label name, size and colour.
4. The colour map contains 166 types, mainly divided into the following four categories: Sequential colormaps: continuous colormaps, Diverging colormaps: discrete colormaps at both ends, Qualitative colormaps: discrete colormaps, Miscellaneous colormaps. Other colormaps. For example, 'Accent', 'Accent_r', 'Blues', 'Blues_r', 'BrBG', 'BrBG_r', etc. Each suffix _r colour map is a horizontal flip of the original colour bar.
5. Number of colour bar scales set the number of scales
6. Bar lable and Bar lable size can set the label and label size of the Color bar.
7. Title, X-lable, Y-lable, Z-lable can set the label.

3. Once all configurations are completed, click the "Run" button to display or update the heat map, which will appear in the canvas on the left.

4. Users can export the plotter by right clicking on the 'Save' export option. Four image formats are available: png, jpg, pdf and svg to suit different needs.

## **3.2.1.1 3D cluster by Row**

The 3D cluster by Row performs column clustering on top of the 3D plotter. Seven clustering methods and 22 distance metrics are provided.

**Select**

Menu -> Expression analysis -> 3D plotter -> 3D heatmap -> 3D cluster by Row

**Input**

Csv format or Excel format target series file


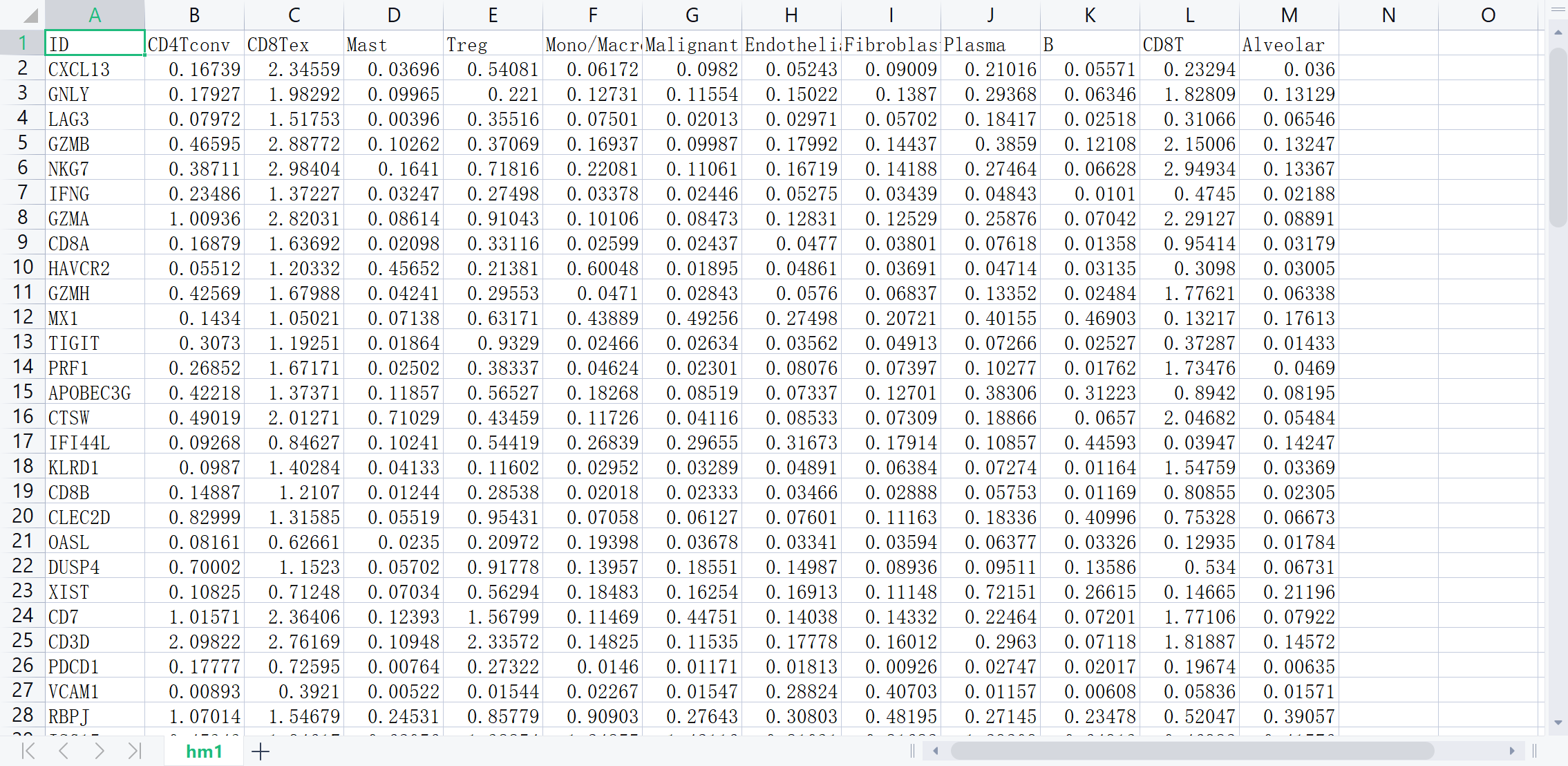


Fig: Input file.

**Output**

Generate image in the left canvas


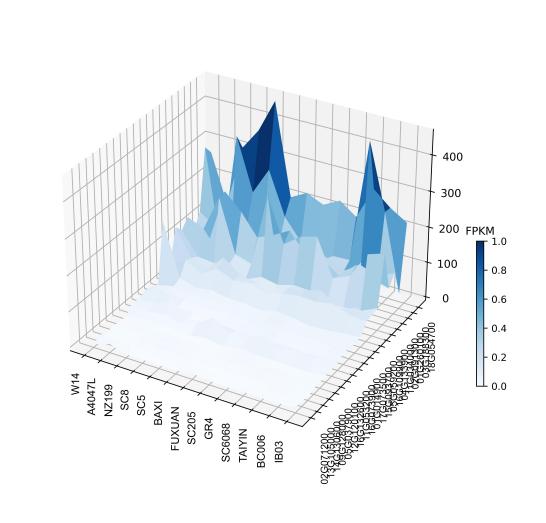


Fig: 3D cluster by Row output image

**Detailed instructions for use**

1. First of all, click the file button on the right, select the Csv format or Excel format file that needs to be visualized.

2. The default parameters have been set, if you want to adjust the parameters please see the following introduction.

1. X-axis, Y-axis, Z-axis set the X-axis, Y-axis, Z-axis on and off, font, font size, colour, rotation angle.
2. Metric can choose 7 types of clustering methods.
3. Method allows you to choose 22 distance metrics.
4. Color bar sets its direction, label font, label name, size and colour.
5. The colour map contains 166 types, mainly divided into the following four categories: Sequential colormaps: continuous colour maps, Diverging colormaps: colour maps with dispersion at both ends, Qualitative colormaps: discrete colour maps, Miscellaneous colormaps. Other colormaps. For example, 'Accent', 'Accent_r', 'Blues', 'Blues_r', 'BrBG', 'BrBG_r', etc. Each suffix _r colour map is a horizontal flip of the original colour bar.
6. Number of colour bar scales set the number of scales
7. Bar lable and Bar lable size can set the label and label size of the Color bar
8. Title, X-lable, Y-lable, Z-lable can set the label

3. Once all configurations are completed, click the "Run" button to display or update the heat map, which will appear in the canvas on the left.

4. The user can export the plotter by right clicking on the 'Save' export option, DataColor offers 900-1000dpi images for the user to use. Four image formats are available: png, jpg, pdf and svg to suit different needs.

## **3.2.1.2 3D cluster by Col**

The 3D plotter (Cluster by Col) performs row clustering on top of the 3D plotter. Seven clustering methods and 22 distance metrics are provided.

**Select**

Menu -> Expression analysis -> 3D plotter -> 3D heatmap -> 3D cluster by Row

**Input**

Csv format or Excel format target series file


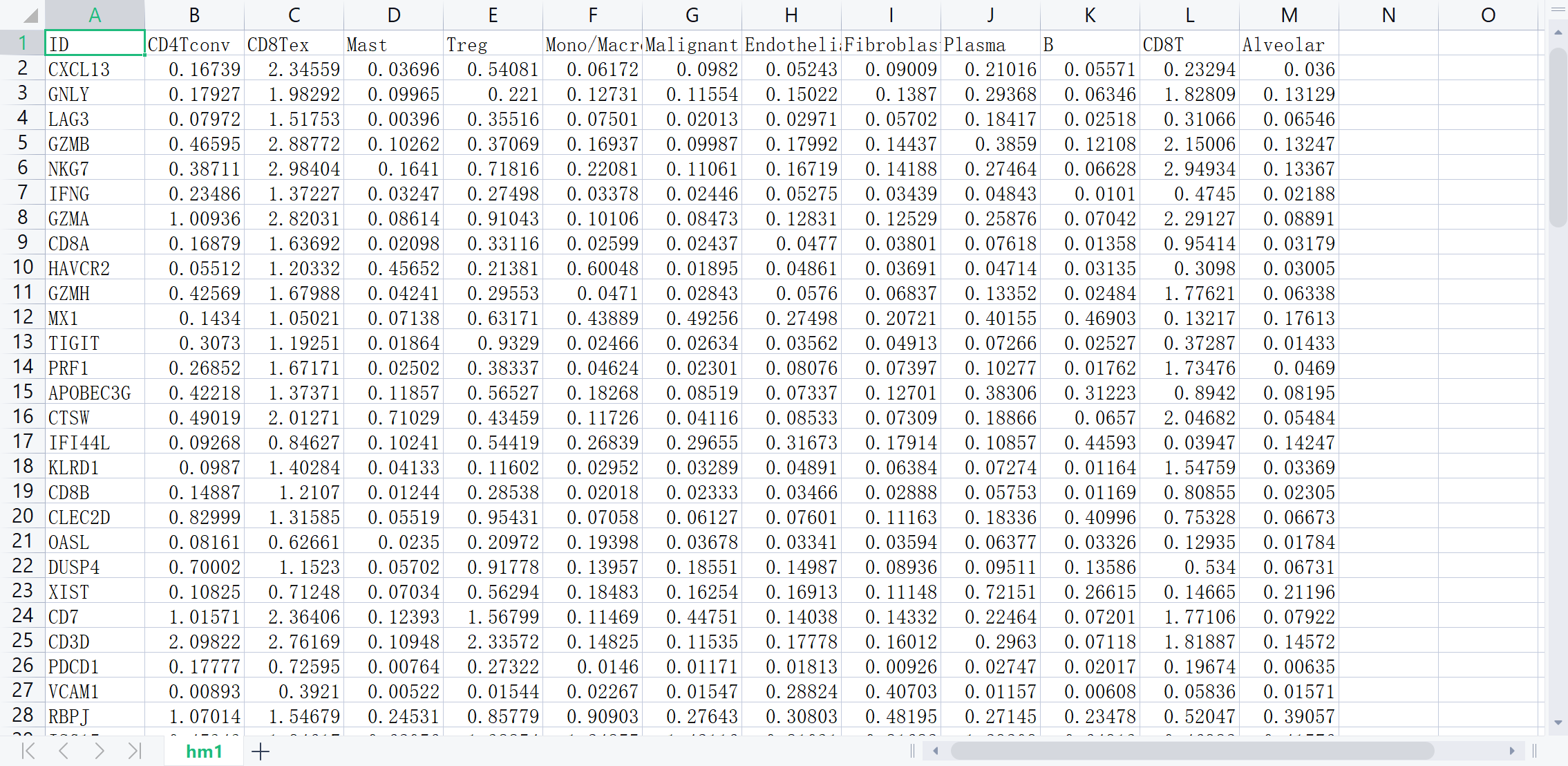


Fig: Input file.

**Output**

Generate image in the left canvas


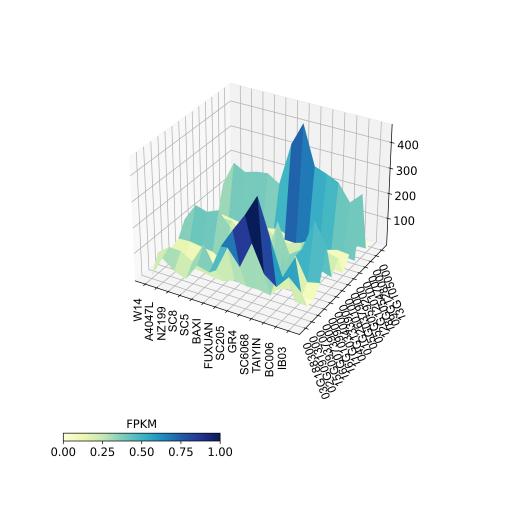


Fig: 3D cluster by Col output image

**Detailed instructions for use**

1. First of all, click the file button on the right, select the Csv format or Excel format file that needs to be visualized.

2. Default parameters have been set, to adjust parameters see 3D cluster by Col parameters.

3. Once all configurations are completed, click the "Run" button to display or update the heat map, which will appear in the canvas on the left.

4. Users can export the plotter by right clicking on the 'Save' export option. Four image formats are available: png, jpg, pdf and svg to suit different needs.

## **3.2.2 3D scatter plotter**

The 3D Scatter plotter is very similar to the 3D plotter in that all data is represented by a square scatter in 3D coordinates and the colour is represented by the corresponding data in 2D. The most characteristic feature is that the 3D plotter when viewed from an overhead perspective is extremely similar to the 2D plotter presentation, ensuring the accuracy of the plotter. The third data can be represented both by colour differences and by the height in 3D space.

**Select**

Menu -> Expression analysis -> 3D plotter -> 3D scatter plotter

**Input**

Csv format or Excel format target series file


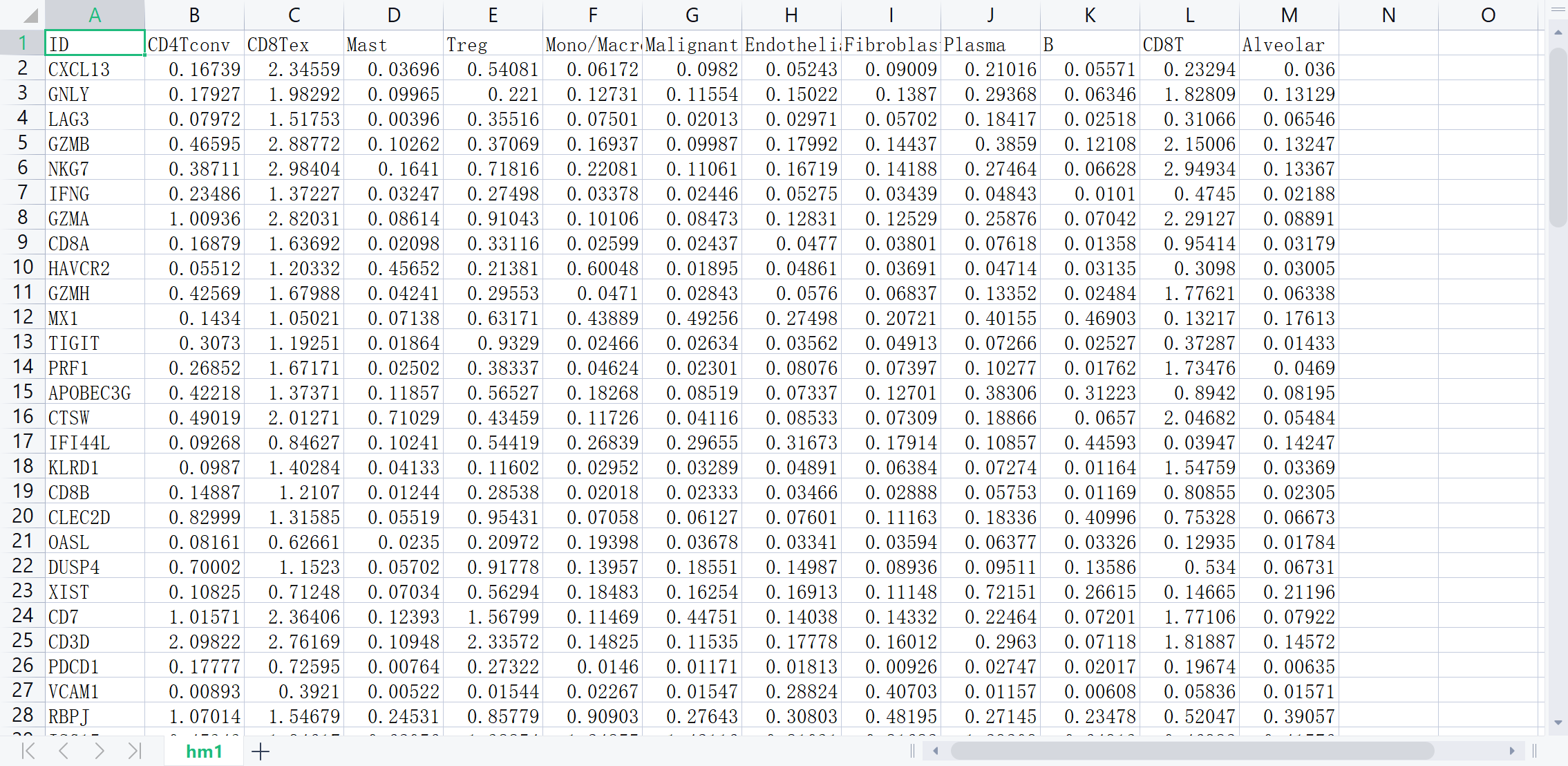


Fig: Input file.

**Output**

Generate image in the left canvas


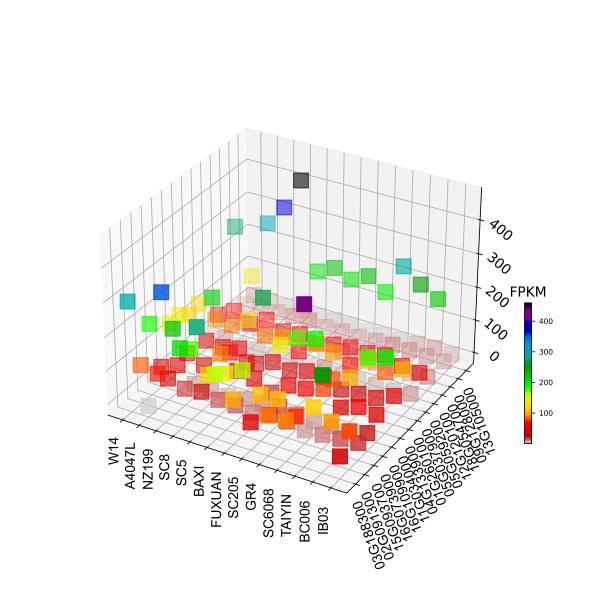


Fig: 3D scatter plotter output image

**Detailed instructions for use**

1. First of all, click the file button on the right, select the Csv format or Excel format file that needs to be visualized.

2. Default parameters have been set, to adjust parameters please see 3D plotter parameters.

3. Once all configurations are completed, click the "Run" button to display or update the heat map, which will appear in the canvas on the left.

4. Users can export the plotter by right clicking on the 'Save' export option. Four image formats are available: png, jpg, pdf and svg to suit different needs.

## **3.2.3 3D Bar plotter**

The 3D Bar plotter, much like the 3D Scatter plotter, represents all data as columns in 3D coordinates, with the colours being represented by their 2D counterparts, the data being represented both by colour differences and by the height of the columns.

**Select**

Menu -> Expression analysis -> 3D plotter -> 3D Bar plotter]

**Input**

Csv format or Excel format target series file


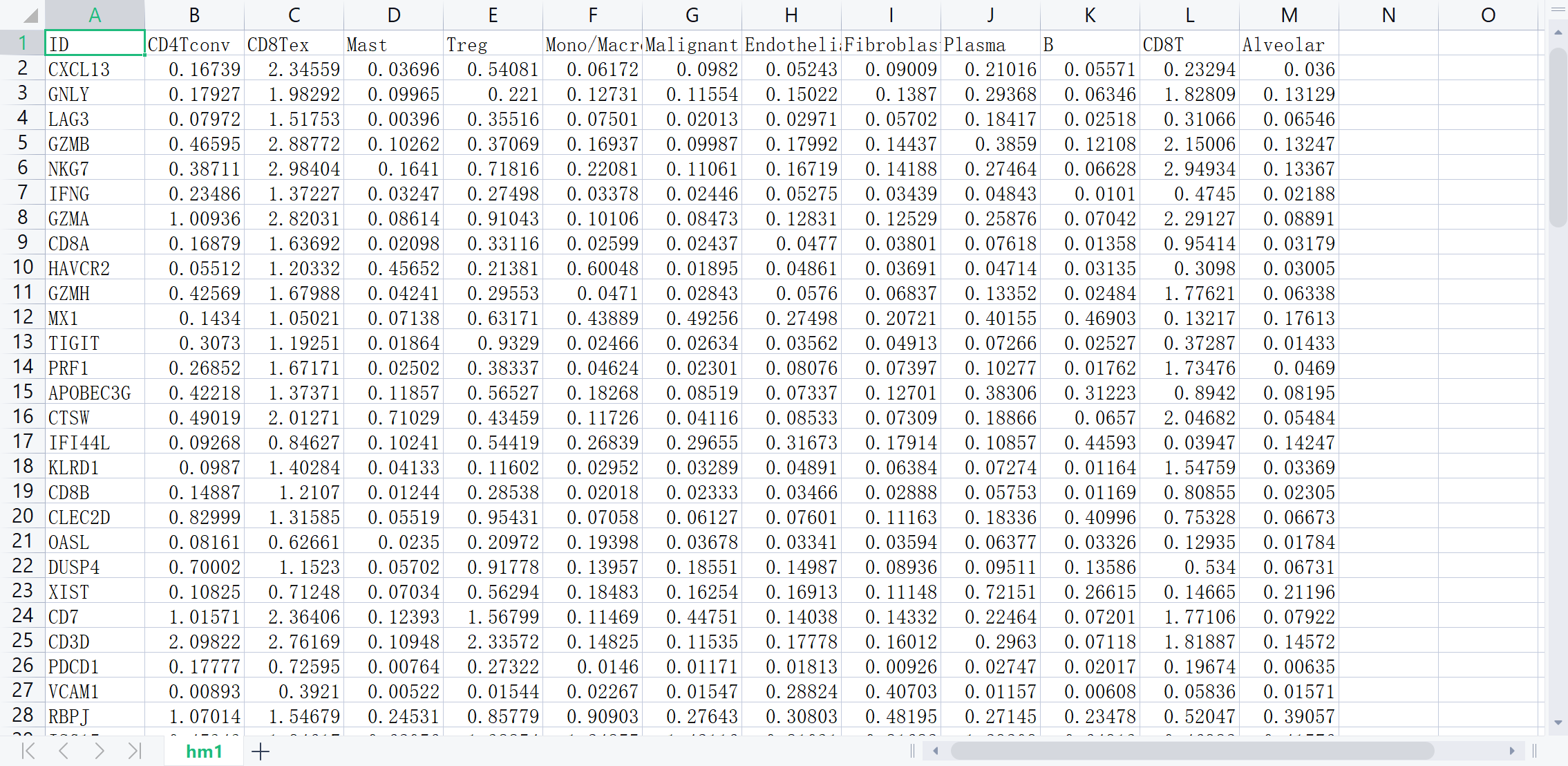


Fig: Input file.

**Output**

Generate image in the left canvas


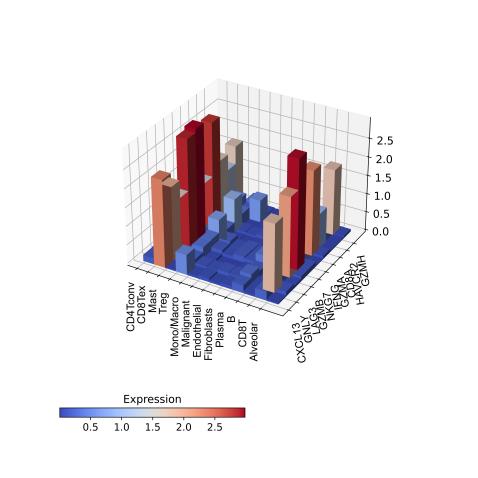


Fig: 3D Bar plotter output image

**Detailed instructions for use**

1. First of all, click the file button on the right, select the Csv format or Excel format file that needs to be visualized.

2. Default parameters have been set, to adjust parameters please see 3D plotter parameters.

3. Once all configurations are completed, click the "Run" button to display or update the heat map, which will appear in the canvas on the left.

4. Users can export the plotter by right clicking on the 'Save' export option. Four image formats are available: png, jpg, pdf and svg to suit different needs.

3.3 Isoheight plotter

3.3.1 Isoheight plotter

Isoheight plotter (Non-cluster) is a plotter model that we have created by combining contour maps with tree clustering. Isoheight plotters use a two-dimensional format to represent three-dimensional data. This type of plotter is better suited to large amounts of biological data, and the differences in colour allow us to see the differences between data at a macro level.

**Select**

Menu -> Expression analysis -> Isoheight plotter -> Isoheight plotter

**Input**

Csv format or Excel format target series file


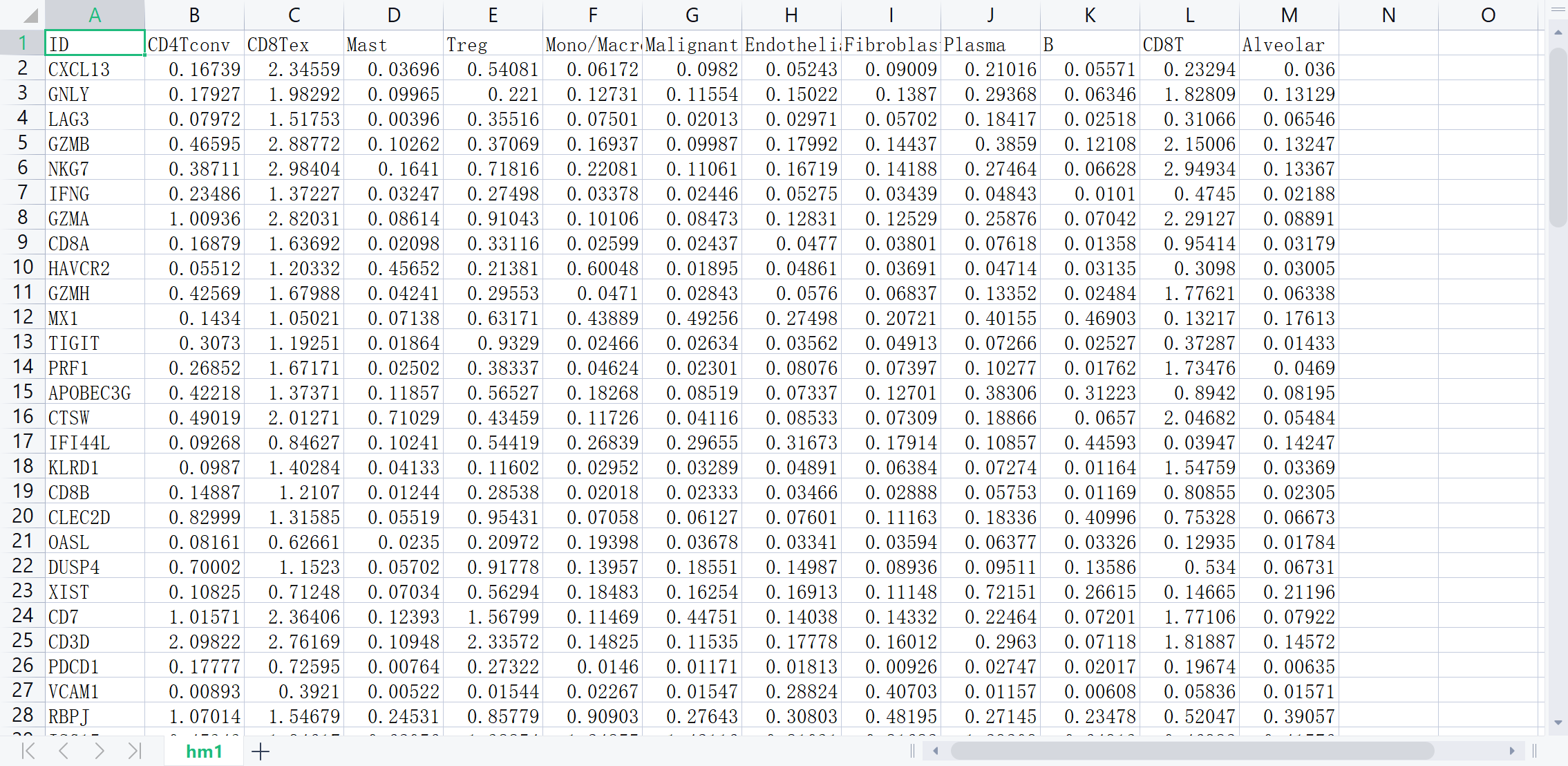


Fig: Input file.

**Output**

Generate image in the left canvas


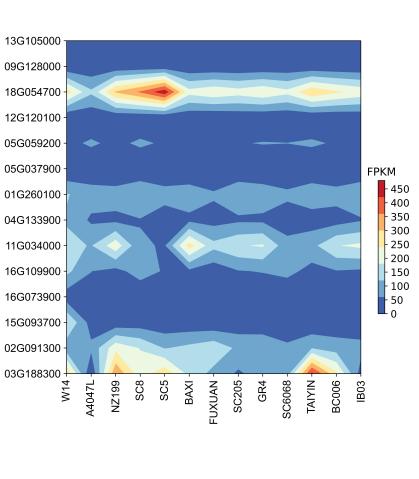


Fig: Isoheight plotter output image

**Detailed instructions for use**

1. First of all, click the file button on the right, select the Csv format or Excel format file that needs to be visualized.

2. The default parameters have been set, if you need to adjust the parameters, please see the introduction below

1. X-axis and Y-axis set the X-axis and Y-axis on and off, font, font size, color, rotation angle and step size.
2. The Cluster by parameter is a clickable button, which will jump to the corresponding clustering tool.
3. Row cluster and Col cluster control the opening and closing of the clustering dendrogram of rows and columns.
4. Scale-min and Scale-max can customize the total value range of the Color bar, and Scale interval sets the value range of each color block on the Color bar, and can change the fineness of the color to adjust the drawing effect.
5. Show data can display the data value of each grid on the heat map, and can also adjust the display font size of the data value.
6. Color bar sets its direction, label font size, label name, size and color.
7. There are 166 color maps, which are mainly divided into the following four categories: Sequential colormaps: continuous color maps, Diverging colormaps: color maps that diverge at both ends, Qualitative colormaps: discrete color maps, Miscellaneous colormaps: other color maps. Such as 'Accent', 'Accent_r', 'Blues', 'Blues_r', 'BrBG', 'BrBG_r', etc., each suffix _r color map is the original color bar flipped horizontally.
8. Number of color bar scales Set the number of scales.
9. Bar lable and Bar lable size can set the label and label font size of Color bar.

3. After completing all configurations, click the "Run" button to display or update the plotter, and the plotter will appear in the left canvas.

4. Users can right-click the "Save" export option to export heat maps, and DataColor provides 900-1000dpi images for users to use. There are four image formats: png, jpg, pdf, and svg, to meet different needs.

## **3.3.2 Isoheight plotter (Cluster)**

Isoheight plotter is a heat map mode that we combine contour map and dendritic clustering. The contour heat map uses two-dimensional form to reflect three-dimensional data. This type of heat map is more suitable for a large amount of biological data, and the high expression value is surrounded by clustering. Through the color difference, we can see the difference between the data macroscopically . The clustering effect is reflected by a variety of tree colors, which is also one of the highlights of the contour heat map.

**Select**

Menu -> Expression analysis -> Isoheight plotter -> Isoheight plotter (Cluster)

**Input**

Csv format or Excel format target series file


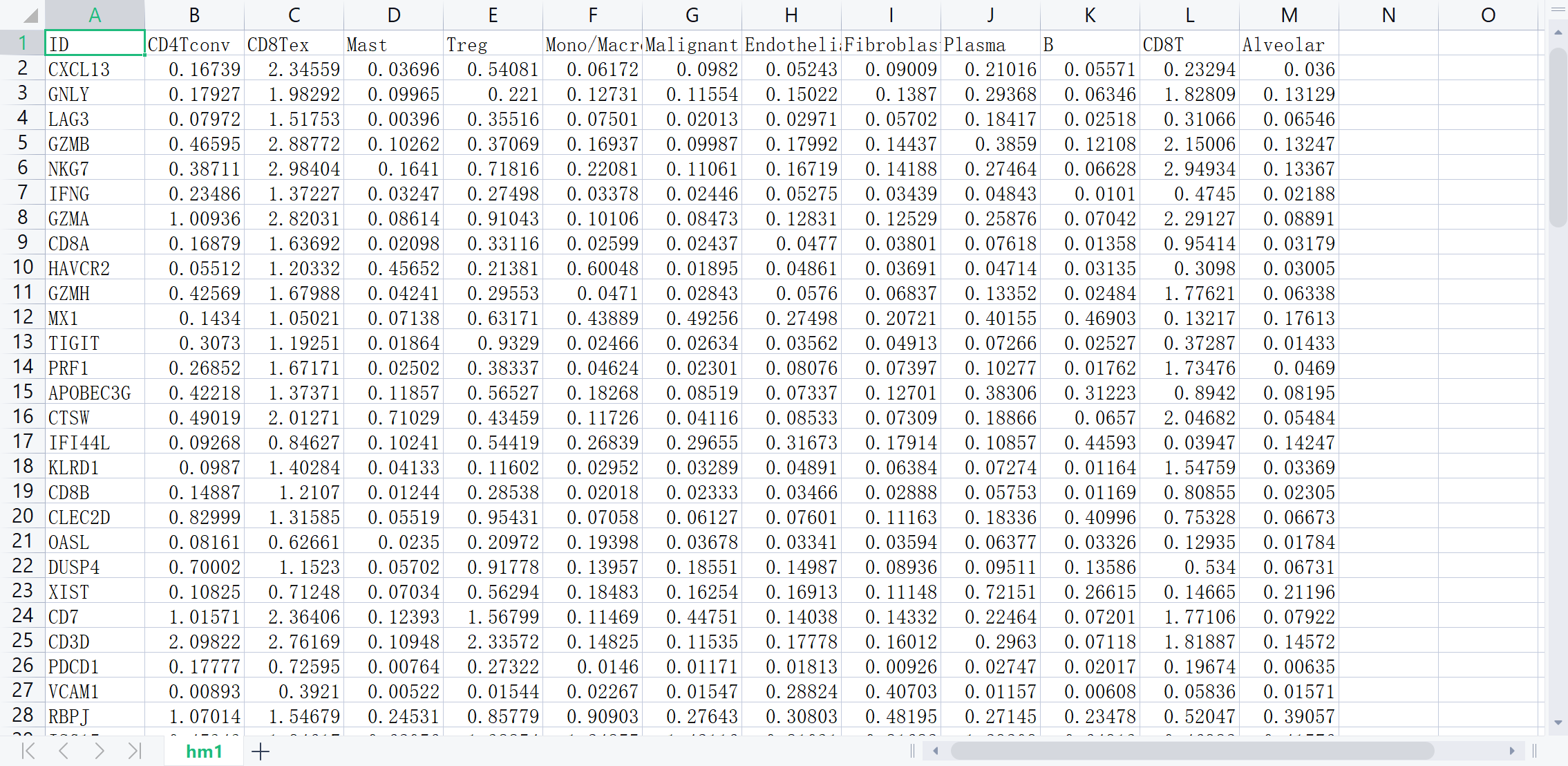


Fig: Input file.

**Output**

Generate image in the left canvas


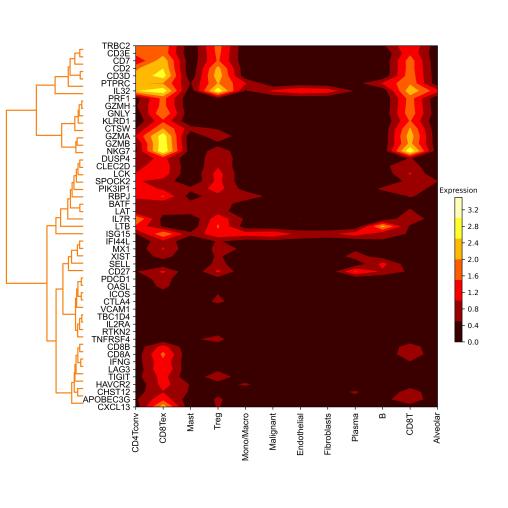


Fig: Isoheight plotter (Cluster) output image

**Detailed instructions for use**

1. First of all, click the file button on the right, select the Csv format or Excel format file that needs to be visualized.

2. The default parameters have been set, if you need to adjust the parameters, please see the introduction below

1. X-axis and Y-axis set the X-axis and Y-axis on and off, font, font size, color, rotation angle and step size.
2. Row cluster and Col cluster control the opening and closing of the clustering dendrogram of rows and columns.
3. Scale-min and Scale-max can customize the total value range of the Color bar, and Scale interval sets the value range of each color block on the Color bar, and can change the fineness of the color to adjust the drawing effect.
4. Show data can display the data value of each grid on the heat map, and can also adjust the display font size of the data value.
5. Metric can choose 7 clustering methods.
6. Method can choose 22 distance measures.
7. Color bar sets its direction, label font size, label name, size and color.
8. There are 166 color maps, which are mainly divided into the following four categories: Sequential colormaps: continuous color maps, Diverging colormaps: color maps that diverge at both ends, Qualitative colormaps: discrete color maps, Miscellaneous colormaps: other color maps. Such as 'Accent', 'Accent_r', 'Blues', 'Blues_r', 'BrBG', 'BrBG_r', etc., each suffix _r color map is the original color bar flipped horizontally.
9. Number of color bar scales Set the number of scales.
10. Bar lable and Bar lable size can set the label and label font size of Color bar.

3. After completing all configurations, click the "Run" button to display or update the plotter, and the plotter will appear in the left canvas.

4. Users can right-click the "Save" export option to export heat maps, and DataColor provides 900-1000dpi images for users to use. There are four image formats: png, jpg, pdf, and svg, to meet different needs.

## **4 Functional omics**

## 4.1 Anatomogram plotter

Displaying tissue information effectively in multicellular organisms can be a time-consuming and laborious process. With different colours representing the expression of tissues in anatomical maps, it is easy to spot differences between tissues or tissues and immediately provide the biological context for these observations and grasp the visualisation results more quickly.

**Select**

Menu+-> Functional omics -> -> Anatomogram plotter

**Input**

Csv format or Excel format target series file


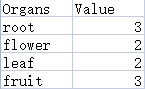


Fig: Input file.

**Output**

Generate image in the left canvas


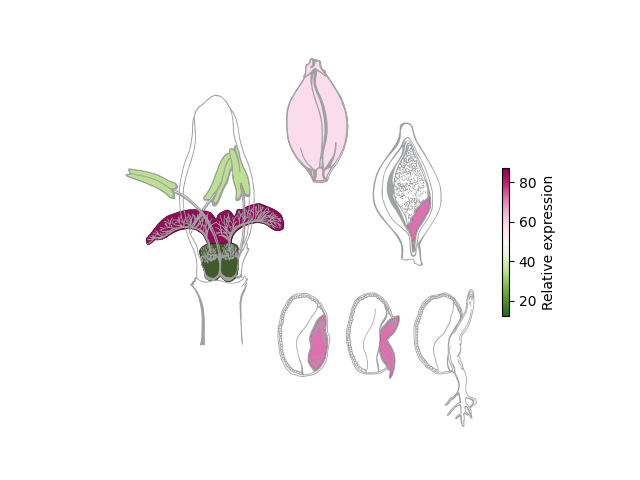


Fig: Anatomogram plotter output image

**Detailed instructions for use**

1. First of all, click the file button on the right, select the Csv format or Excel format file that needs to be visualized.

2. The default parameters have been set, if you need to adjust the parameters, please see the introduction below

1. Type selects the desired base map for the deconvolution map
2. The colour map contains 166 types, mainly divided into the following four categories: Sequential colormaps: continuous colour maps, Diverging colormaps: colour maps with dispersion at both ends, Qualitative colormaps: discrete colour maps, Miscellaneous colormaps. Other colormaps. For example, 'Accent', 'Accent_r', 'Blues', 'Blues_r', 'BrBG', 'BrBG_r', etc. Each suffix _r colour map is a horizontal flip of the original colour bar.
3. Color bar sets its orientation selection.
4. Title, table, Color bar can set the label name.

3. Once all configurations are complete, click the 'Run' button to display or update the Anatomogram plotter, which will appear in the canvas on the left.

4. The user can export the heat map by right clicking on the 'Save' export option, DataColor offers 900-1000 dpi images for the user to use. Four image formats are available: png, jpg, pdf and svg to suit different needs.

## 4.2 Enrichment bar plotter

Enrichment bar plotter is a multivariate histogram. Through the position, height and color size of the columns, the correlation between data can be analyzed. It is suitable for data with small amount of data and clear location relationship. GO and KEGG enrichment data are common usage scenarios of Enrichment bar plotterr. The color of the bar represents the p value (or q value, etc.), and the size represents the number of genes.

**Select**

Menu -> Functional omics -> Enrichment bar plotter

**Input**

Csv format or Excel format target series file

.
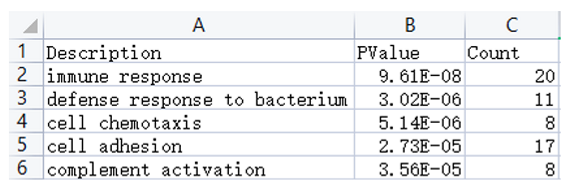


Fig: Input file.

**Output**

Generate image in the left canvas


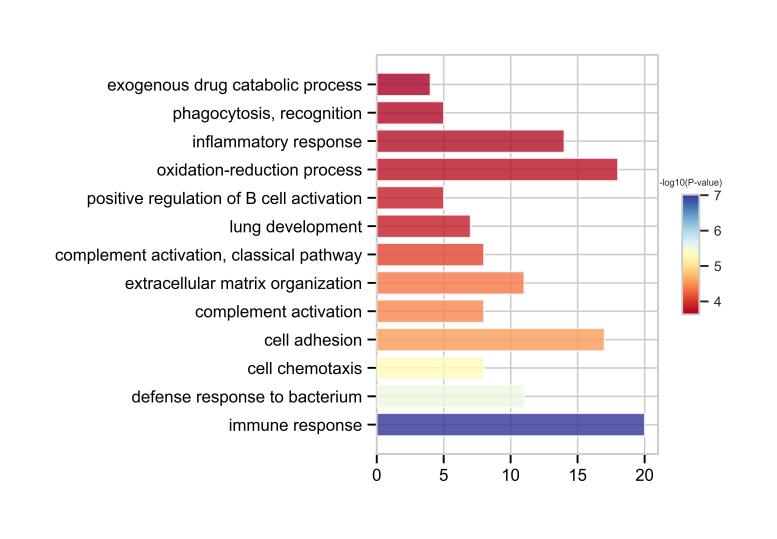


Fig: Enrichment bar plotter output image

**Detailed instructions for use**

1. First of all, click the file button on the right, select the Excel format or Csv format file that needs to be visualized
2. Default parameters have been set, if you want to adjust the parameters, please see the following introduction

## Set the X-axis and Y-axis on and off, font, font size, color, rotation angle and step length

## Color bar sets its direction, label size, label name, size, color, position, font size.

## color map contains 166 kinds, mainly divided into the following four categories: Sequential colormaps: continuous color maps, Diverging colormaps: color maps with dispersion at both ends, Qualitative colormaps: discrete color maps, Miscellaneous colormaps: Other colormaps. Such as 'Accent', 'Accent_r', 'Blues', 'Blues_r', 'BrBG', 'BrBG_r', etc. Each suffix _r color map is a horizontal flip of the original color bar.

## Transparency parameter can adjust the transparency of the dot color.

## 3. After finishing all the configurations, click "Run" button to display or update Bubble plot, Bubble plot will appear in the left canvas.

## 4. Users can export the heat map by right-clicking the "Save" export option, and DataColor provides 6 levels of images from 300-1000 dpi for users to use. There are four image formats: png, .jpg, pdf, and .svg, to meet different needs.

## 4.3 Bubble plotter

Bubble plotter is a multivariate chart and a variant of scatter plot. Bubble plot is a combination of scatter plot and percentage area plot. bubble plot can analyze the correlation between data by the position, area and color size of bubbles. GO and KEGG enrichment data are common scenarios for Bubble plot, where the color of the bubble indicates the p-value (or q-value, etc.) and the size indicates the number of genes.

**Select**

Menu -> Functional omics -> Bubble plotter

**Input**

Csv format or Excel format target series file

.
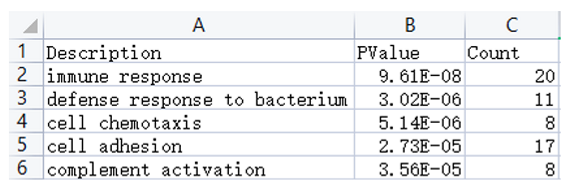


Fig: Input file.

**Output**

Generate image in the left canvas


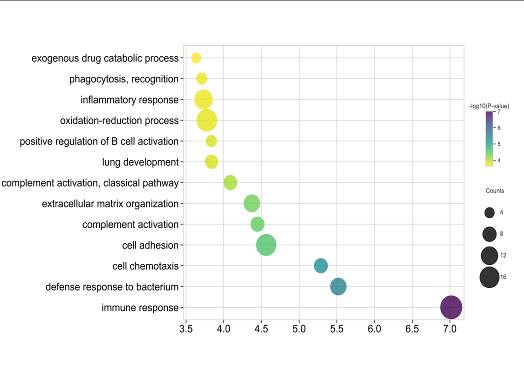


Fig: Bubble plotter output image

**Detailed instructions for use**

1. First of all, click the file button on the right, select the Excel format or Csv format file that needs to be visualized
2. Default parameters have been set, if you want to adjust the parameters, please see the following introduction

## Set the X-axis and Y-axis on and off, font, font size, color, rotation angle and step length

## Color bar sets its direction, label size, label name, size, color, position, font size.

## Bar lable2 is used as the label of size bar, and Bar lable2 size parameter is added.' Distance between entries in the legend' parameter can set the size bar internal label distance

## color map contains 166 kinds, mainly divided into the following four categories: Sequential colormaps: continuous color maps, Diverging colormaps: color maps with dispersion at both ends, Qualitative colormaps: discrete color maps, Miscellaneous colormaps: Other colormaps. Such as 'Accent', 'Accent_r', 'Blues', 'Blues_r', 'BrBG', 'BrBG_r', etc. Each suffix _r color map is a horizontal flip of the original color bar.

## Dot shape sets 15 kinds of dot shapes.

## Dot size magnification can adjust the size of the dot.

## Transparency parameter can adjust the transparency of the dot color.

## 3. After finishing all the configurations, click "Run" button to display or update Bubble plot, Bubble plot will appear in the left canvas.

## 4. Users can export the heat map by right-clicking the "Save" export option, and DataColor provides 6 levels of images from 300-1000 dpi for users to use. There are four image formats: png, .jpg, pdf, and .svg, to meet different needs.

## 4.4 Dot plotter

Datacolor adds Dotplot function, which can use point, diamond, circle, star and other 23 different shapes to draw heat map, increasing the richness of heat map graphic display, except the color represents the data, where the size of the dot and the shade of the color are the same, while representing the size of the value. And you can achieve synchronous zooming in and out of points.

**Select**

Menu -> Functional omics -> Dotplot

**Input**

Csv format or Excel format target series file

.
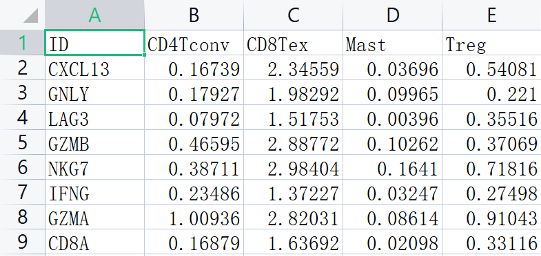


Fig: Input file.

**Output**

Generate image in the left canvas


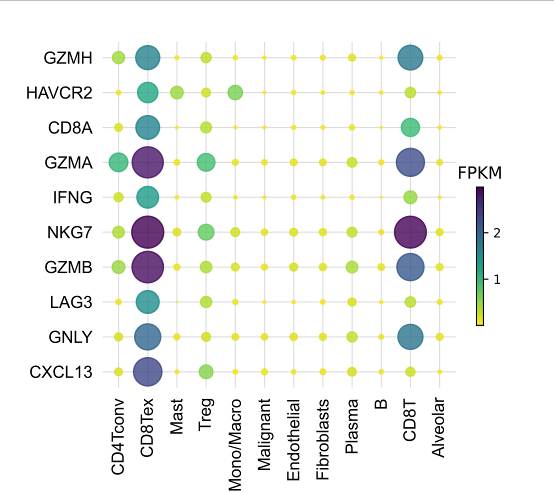


Fig: Dotplot output image

**Detailed instructions for use**

## 1. First of all, click the file button on the right, select the Csv format or Excel format file that needs to be visualized

## 2. Default parameters have been set, if you need to adjust the parameters, please see below.

## Detailed use method

## Set the X-axis and Y-axis on and off, font, font size, color, rotation angle and step length.

## Color bar sets its direction, label size, label name, size, color, position, font size.

## The color map contains 166 types, mainly divided into the following four categories: Sequential colormaps: continuous color maps, Diverging colormaps: discrete color maps at both ends, Qualitative colormaps: discrete color maps, Miscellaneous colormaps: Other colormaps. Such as 'Accent', 'Accent_r', 'Blues', 'Blues_r', 'BrBG', 'BrBG_r', etc. Each suffix _r color map is a horizontal flip of the original color bar.

## Dot shape sets 23 kinds of dot shapes.

## Dot size magnification can adjust the size of the dot.

## Transparency parameter can adjust the transparency of the dot color.

## 3. After finishing all the configurations, click the "Run" button to display or update the heat map, which will appear in the canvas on the left.

## 4. Users can export the heat map by right-clicking the "Save" export option, and DataColor provides 6 levels of images from 300-1000dpi for users to use. There are four image formats: png, .jpg, pdf, and .svg, to meet different needs.

## 4.5 Network plotter

Network plot function is a graphical model that is composed of two factors: connecting lines and nodes. different nodes that are linked, connected by 1 or more lines, and plotting the node size according to the number of correlations. dataColor adds six different ways of node layout, including random, graphical and algorithmic layout, to give users a better custom node experience. It is often used in differential co-expression network analysis to show the correlation of different genes.

**Select**

Menu -> Functional omics -> Network plot

**Input**

Csv format or Excel format target series file


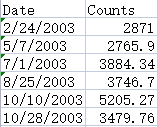


Fig: Input file.

**Output**

Generate image in the left canvas


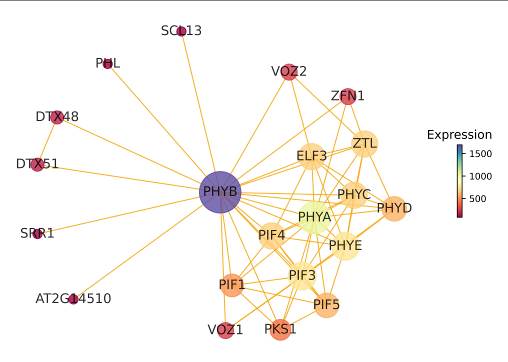


Fig:Network plot output image

**Detailed instructions for use**

1. First of all, click the file button on the right, select the Excel format or Csv format file that needs to be visualized

2. Have set the default parameters, if you need to adjust the parameters, please see the following introduction

1. Set the font size and color of the node.
2. Color bar sets its direction, label font size, label name, size, color, position, font size.
3. Color map contains 166 kinds, mainly divided into the following four categories: Sequential colormaps: continuous color maps, Diverging colormaps: color maps with dispersion at both ends, Qualitative colormaps: discrete color maps, Miscellaneous colormaps: Other colormaps. Such as 'Accent', 'Accent_r', 'Blues', 'Blues_r', 'BrBG', 'BrBG_r', etc. Each suffix _r color map is a horizontal flip of the original color bar.
4. Nodes size magnification can adjust the size of the points.
5. Transparency parameter can adjust the transparency of the point color.
6. Nodes arrangement parameter adds six different node layout methods, including random, graphical and algorithmic layout, etc.

3. After finishing all the configurations, click "Run" button to display or update the Network plot, which will appear in the canvas on the left.

4. Users can export the heat map by right-clicking the "Save" export option, and DataColor provides 6 levels of images from 300-1000 dpi for users to use. There are four image formats: png, .jpg, pdf, and .svg, to meet different needs.

## 4.6 Treemap

Treemap diagrams, also known as rectangular tree mapping, rectangular tree mapping and rectangular tree mapping, refer to a method of displaying tree data using nested rectangles. This method of presentation allows different categories to be presented in different coloured blocks and allows the size of the blocks to be compared to the size of each category. The larger the range of the blocks, the larger and more numerous the category.

**Select**

Menu -> Functional omics -> Treemap

**Input**

Csv format or Excel format target series file


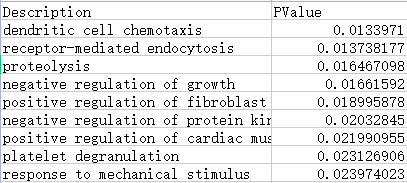


Fig: Input file.

**Output**

Generate image in the left canvas


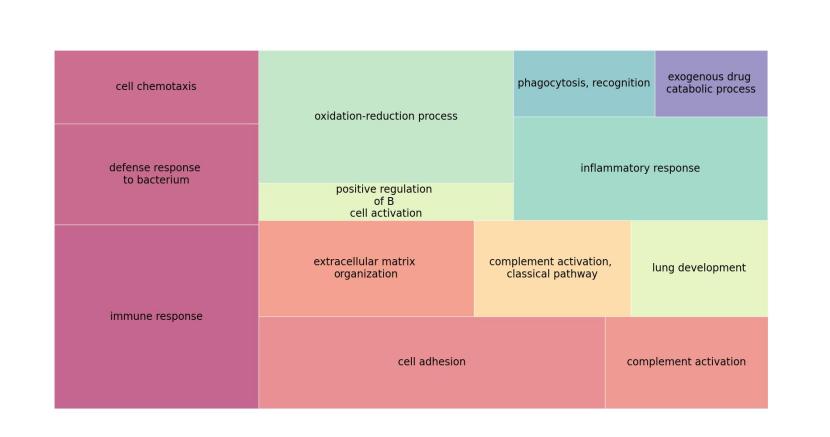


Fig: Treemap output image

**Detailed instructions for use**

1.First of all, click the file button on the right, select the Csv format or Excel format file that needs to be visualized.

2. The default parameters have been set, if you need to adjust the parameters, please see the introduction below

1. Show lable displays the data values of each square on the Treemap and allows you to adjust the display size of the lable.
2. The colour map contains 166 types and is divided into four main categories: Sequential colormaps: continuous colormaps, Diverging colormaps: discrete colormaps at both ends, Qualitative colormaps: discrete colormaps, Miscellaneous colormaps. Other colormaps. For example, 'Accent', 'Accent_r', 'Blues', 'Blues_r', 'BrBG', 'BrBG_r', etc. Each suffix _r colour map is a horizontal flip of the original colour bar.
3. Border widths are the dividing lines for each cell of the Treemap, if you need to adjust the colour, select a value greater than 0 for the width.
4. Transparency sets the transparency of the colour bar, ranging from 0 to 1.
5. Title sets the label and font size.

3. After completing all configurations, click the 'Run' button to display or update the Treemap, which will appear in the canvas on the left.

4. Users can right-click the "Save" export option to export heat maps, and DataColor provides 900-1000dpi images for users to use. There are four image formats: png, jpg, pdf, and svg, to meet different needs.

## 4.7 Wordcloud plotter

A Wordcloud, also known as a word cloud, is a visual representation of text data, consisting of a cloud-like coloured graph of words, used to display large amounts of text data. The importance of each word is displayed in font size or colour.

The Wordcloud is mainly used for frequency analysis of keyword occurrences in text content and is suitable for visualisation of text content mining. Words that appear more frequently in the word cloud are presented in a larger format and words that appear less frequently are presented in a smaller format. The word cloud is essentially a point graph, the result of drawing text with a specific style at the corresponding coordinate points.

**Select**

Menu -> Functional omics -> Wordcloud plotter

**Input**

Csv format or Excel format target series file


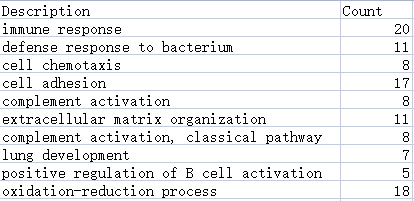


Fig:Input file.

**Output**

Generate image in the left canvas


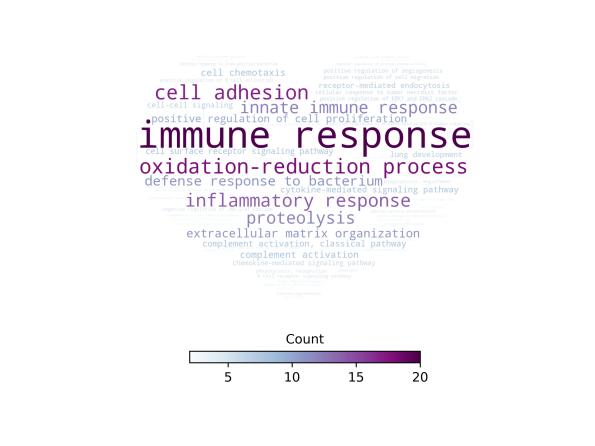


Fig: Wordcloud plotter output image

**Detailed instructions for use**

1. First of all, click the file button on the right, select the Csv format or Excel format file that needs to be visualized.

2. The default parameters have been set, if you need to adjust the parameters, please see the introduction below

1. Width and Height set the width and height of the output image.
2. Background colour parameter selects the colour of the background
3. Word distance sets the distance between words.
4. Maximum word count and maximum font size allows you to customise the total range of font size values.
5. Colour map contains 166 types, mainly divided into the following four categories: Sequential colormaps: continuous colormaps, Diverging colormaps: discrete colormaps, Qualitative colormaps: discrete colormaps, Miscellaneous colormaps. Other colormaps. For example, 'Accent', 'Accent_r', 'Blues', 'Blues_r', 'BrBG', 'BrBG_r', etc. Each suffix _r colour map is a horizontal flip of the original colour bar.
6. 'The color is the same as the background image color' asks if the output image font colour is the same as the background image.

3. Once all configurations are complete, click the 'Run' button to display or update the Wordcloud Map, which will appear in the canvas on the left.

4. The user can export the heat map by right clicking on the 'Save' export option, DataColor offers 900-1000 dpi images for the user to use. Four image formats are available: png, jpg, pdf and svg to suit different needs.
